# Effective coverage and associated factors of antenatal care service among women who attended antenatal care at West Gojjam Zone Public Hospitals, Northwest Ethiopia

**DOI:** 10.1371/journal.pone.0347341

**Published:** 2026-04-21

**Authors:** Bekalu Endalew, Tsehay Awoke Kassa, Muluye Gebrie Mengie, Dejen Tsegaye Alem, Yihalem Abebe Belay

**Affiliations:** 1 Department of Public Health, College of Medicine and Health Sciences, Debre Markos University, Debre Markos, Ethiopia; 2 Bahir Dar Zuria District Health Office, North Gojjam Zone, Bahir Dar, Ethiopia; 3 Department of Nursing, College of Medicine and Health sciences, Debre Markos University, Debre Markos, Ethiopia; 4 Graduate school of medicine, Faculty of Science, Medicine and Health, University of Wollongong, Wollongong, Australia; Arba Minch University, ETHIOPIA

## Abstract

**Background:**

Effective coverage reflects the performance of a health system by integrating need, service utilization, and quality of care into a single measure. In many developing countries, inadequate effective coverage of antenatal care services along with poor quality maternal health services and low levels of awareness about healthcare has contributed to maternal mortality ratios that are 14 times higher than those in developed countries. Despite this significant disparity, there is limited evidence available regarding the effective coverage of antenatal care in Ethiopia. Therefore, this study aimed to assess the effective coverage of antenatal care and associated factors among women who attended antenatal care at West Gojjam Zone Public Hospitals, northwest Ethiopia.

**Methods:**

A facility-based retrospective cross-sectional study design was carried out in West Gojjam public hospitals from June 30 to July 30, 2023. Study participants were selected using a multi-stage simple random sampling technique using patient identification number as a sampling frame. Data were gathered from hospital health information data systems and client charts, entered into Epi data version 4.6, and then exported to SPSS statistical package version 25 for further analysis. Both bi-variable logistic regression and multi-variable logistic regression analyses were carried out. Variables with p-value <0.25 in the bi variable logistic regression were entered into multi-variable logistic regression. Variables having P-values <0.05 in the multi-variable logistic regression were considered as statistically significant predictors of the outcome variable.

**Results:**

The effective coverage of antenatal care service was 25.9% (95% CI: 22.6, 29.4%) in the study area. Age of mothers ranged 25–34 (AOR: 0.32; 95% CI; 0.20–0.50), wanted pregnancy (AOR: 1.65; 95% CI; 1.08–2.55), presence of previous obstetric complication (AOR: 6.27; 95% CI; 3.83–10.28) and presence of previous gynecological complication (AOR: 2.08; 95% CI; 1.66–3.77) were statistically significantly associated variables with effective coverage of antenatal care services.

**Conclusion:**

In this study one out of four mothers received effective coverage of antenatal care in the study area. Age of mothers ranged from 25 to 34, wanted pregnancy, presence of previous obstetric complication and presence of previous gynecological complication were statistical significant predictors of effective coverage of antenatal service. Therefore, boosting effective antenatal care requires targeted interventions based on identified predictors.

## Introduction

Maternal mortality remains unacceptably high and continues to be a major global public health concern [1]. The number of women who die each year from complications of pregnancy and childbirth declined from 443,000 in 2000–260,000 in 2023, despite rapid population growth in many high-burden countries. Nevertheless, an estimated 712 women still die each day from pregnancy and childbirth related complications equivalent to one death every two minutes [2]. Ethiopia continues to face a substantial maternal health challenge, with a maternal mortality ratio of 195 deaths per 100,000 live births. This figure remains considerably higher than the Sustainable Development Goal (SDG) target of fewer than 70 deaths per 100,000 live births by 2030, indicating a significant gap that must be addressed [3].

Antenatal care (ANC) offers a critical opportunity to identify pregnancy-related risk factors and complications and to enhance birth preparedness, thereby reducing maternal and neonatal morbidity and mortality [4,5]. The proportion of women attending four or more ANC visits (ANC 4+) has been widely used as an indicator for monitoring maternal health service utilization and overall health system performance [6,7]. However, contact coverage alone does not ensure that women receive high-quality care, and access to quality services may be inequitable [8]. In low- and middle-income countries (LMICs), even where ANC 4 + coverage is relatively high, wealthier and better-educated women are more likely to receive comprehensive and quality care [9]. This suggests that crude coverage measures may overestimate health system performance, as they fail to capture whether essential interventions are actually delivered. Persistently high maternal mortality may therefore be partly attributed to poor quality of care and inadequate uptake of key interventions during routine ANC visits [10]. To address these limitations, measuring effective coverage (EC) of antenatal care is essential. Effective coverage provides a more comprehensive assessment of health system performance by integrating three interrelated components: need, use, and quality [10]. Need refers to women who require ANC services (i.e., pregnant women); use refers to their actual utilization of services (such as attending at least one or four ANC visits); and quality refers to the extent to which appropriate evidence-based interventions are delivered and translate into potential health benefits. In the context of ANC, effective coverage is typically defined by combining attendance (at least four visits) with the receipt of recommended service content [11]. Standard ANC content includes a set of evidence-based interventions such as screening tests, preventive measures, and timely management provided at specified stages of pregnancy. Receipt of some or all recommended components during at least one visit is commonly used to assess quality of care [12,13]. Factors influencing effective coverage of essential ANC services can be broadly categorized into structural, intermediary, and health system domains. Structural factors include fundamental social determinants such as wealth status, ethnicity, and gender. Intermediary factors encompass living and working conditions, family and community contexts, and individual characteristics that shape health behaviors. Health system factors relate to the organization, availability, and delivery of quality health services [14].

Although existing literature addresses contact coverage of routine maternal and newborn health (MNH) visits [15,16] and perceived quality of care [17,18], but the effective coverage (EC) of routine MNH visits in our region is poorly understood [19]. West Gojjam Zone has been shown to have very low completion of key maternal health services along the continuum of care, with only about 12% of women completing the pathway from ANC attendance to skilled birth attendance and postnatal care, and many women failing to receive recommended antenatal interventions, indicating substantial gaps in both service utilization and quality that warrant focused investigation of effective ANC coverage in this setting [20]. Therefore, the study aimed to assess the effective coverage of antenatal care and identify associated factors among women attending ANC at public hospitals in West Gojjam Zone, Northwest Ethiopia.

## Methods

### Study design and period

A facility-based retrospective cross-sectional study was conducted in West Gojjam public hospitals. Data were collected between June 30 and July 30, 2023, from ANC charts covering the period September 1, 2021, to August 30, 2022.

### Study area settings

West Gojjam is one of the administrative zones of the Amhara Region, located 387 km northwest of Addis Ababa, the national capital, and 176 km northeast of Bahir Dar, the capital of the Amhara Region. The west Gojjam zone projected population is 3,335,515 by 2023, with 1,634,402 men and 1,701,113 women living there [21]

The zone hosts one general hospital and seven primary public hospitals, namely Finote Selam, Burie, Dur Betie, Merawi, Adet, Liben, Feres Bet, and Dembecha. These facilities offer obstetrics and gynecological services, including family planning, antenatal care (ANC), delivery, postnatal care, and various surgical procedures. The average number of ANC4 visits per year at each hospital was as follows: Finote Selam General Hospital (1,197), Burie Primary Hospital (658), Durbete Primary Hospital (364), Merawi Primary Hospital (374), Adet Primary Hospital (590), Liben Primary Hospital (394), Feres Bet Primary Hospital (730), and Dembecha Primary Hospital (742). Services are provided by midwives, obstetricians/Integrated Emergency Surgical Officers, and general practitioners [22].

### Population

The source population consisted of all pregnant women who had ANC follow-up at the Maternal and Child Health (MCH) units in public hospitals of West Gojjam zone, while the study population consisted of all pregnant women who had ANC follow-up at the Maternal and Child Health (MCH) units of selected West Gojjam zone public hospitals during the data collection period. Women who had discontinued their ANC follow-up, referred to another health institution, or had transferred from another institution were excluded from this study.

### Sample size determination

The sample size for both objectives was calculated. The largest sample size was considered, which is calculated using the single population proportion formula, n =$\;({Za/\text{2})}^{\text{2}}(P)(\text{1}-P)/{\left(d\right)}^{\text{2}}$ , where “n” is sample size, “p” is proportion of pregnant women who received effective antenatal care services, “1-p” proportion of pregnant women who did not receive effective antenatal care services and “d” is margin of error with the assumptions of a proportion of 22%; a study conducted in Ethiopia [23], a 95% confidence level, and a 4% margin of error, 1.5 design effect and 10% non-response. Accordingly, the final sample size was 680.

### Sampling procedures

A multi-stage sampling technique was employed to select the study participants. Firstly, three hospitals (38%) were chosen by lottery method out of eight public hospitals in the West Gojjam Zone. Then, based on their preliminary case load data at the selected hospitals, proportional allocation of a sample was applied. Finally, a simple random sampling technique was employed to select the study participants using their medical registration numbers as a sampling frame.

### Variables of the study

Effective coverage of ANC service was the study’s outcome variable. Socio-demographic characteristics (age, marital status and place of residency), obstetrics and gynecology related factors (parity, gravidity, status of pregnancy, previous mode of delivery, abortion, previous history of obstetric complication (premature rupture of membrane, antepartum hemorrhage, pregnancy induced hypertension, postpartum hemorrhage), pervious history of gynecological complication (pelvic inflammatory diseases, adnexal torsion, tumor), investigation and infrastructure related variables (distance from hospital, utilizing of ultrasound, use of baseline investigation for pregnant women, seen by trained human power) were the study’s independent variables.

### Measurements

**Effective coverage of ANC interventions**: For this particular study, pregnant women who had attended four or more antenatal care (ANC) visits and received all the World Health Organization-recommended interventions at least once during their ANC follow-up period were well thought out, as they had received an effective coverage of Antenatal care [24].

**WHO recommended interventions:** Interventions comprehended screening for hypertension, symphysis-fundal height (SFH) measurement, anemia, gestational diabetes mellitus, asymptomatic bacteriuria, Rh-type, and tetanus immunization status, as well as antenatal ultrasound, were considered as the WHO-recommended interventions in this study [24].

**Baseline investigation**: All pregnant women routinely underwent investigations, including hematocrit (HCT), Venereal Disease Research Laboratory (VDRL) testing, blood group and Rh typing (BG/Rh), and urinalysis (U/A), irrespective of risk assessment [25].

**Trained human power:** Maternal and child health (MCH) clinics employed a multidisciplinary team of healthcare professionals, involving certified midwives and integrated emergency surgical officers (IESOs)/obstetricians [26].

**Adequate equipment and materials at MCH/ANC clinic:** Adequate provision of equipment and supplies, including tape measures, fetoscopes, weight scales, height measurement tools, screens, blood pressure apparatuses, thermometers, examination beds, and registration books, is essential for antenatal care (ANC) clinics [26].

**Pregnancy status**: in this study pregnancy status refers to whether the pregnancy is wanted or unwanted. Wanted conception is often measured retrospectively through surveys by asking individuals, “Did you want to become pregnant at all?” If the response is “wanted to become pregnant then,” they were classified as wanted otherwise not wanted [27].

**Abortion:** is the termination of pregnancy before fetal viability, which is conventionally taken to be less than 28 weeks from last normal menstrual period in Ethiopian setup [28].

### Data collection tools and procedures

Data were collected from client charts using structured questionnaire adapted from previous related literatures [23,29–33]. Three-diploma and two BSC nurses were recruited as data collectors and supervisors respectively.

### Data quality control

Data collectors and supervisors took one-day training on the study objectives, data collection instruments, methodologies, and procedures prior to data collection. A pretest was conducted on 34 (5%) pregnant women at Injibara General Hospital, which was outside the study area, and necessary changes were made based on the results of the pre-test. The lead investigator and supervisors reviewed the consistency and completeness of the data daily during the data collection time.

### Data management and analysis

The collected data were coded and entered to Epi data version 4.6 Software, then exported to the SPSS statistical package version 25 for further analysis. Before analysis, data were cleaned for possible errors. Binary logistic regression analysis was carried out to identify candidate variables. Model adequacy was checked using Hosmer and lemeshow test and was well fit to the data. Variables found to be associated with the outcome of interest at p-value <0.25 in the bi-variable logistic regression analysis were entered into the multi-variable logistic regression analysis to control for the possible effect of confounders. This more liberal threshold helps ensure that potentially important predictors or confounders are not excluded prematurely, particularly in studies with moderate sample sizes, such as ours (n = 680), where weaker associations may not reach conventional significance in bi-variable analysis but could be important when adjusted for other variables. This approach is based on the purposeful selection strategy described by Hosmer and Lemeshow, who recommend considering variables with p-values up to 0.25 to avoid excluding potentially relevant covariates in the initial model-building process [34].Multi-colinearity was checked using variance inflation factor (VIF) and tolerance test and there was no multi-colinearity between independent factors. Having a p value, less than 0.05 with 95% confidence intervals in the multivariable logistic regression analysis was used to declare the statistically significant association between the predictor variables and the outcome variable.

### Ethical considerations

Ethical clearance was obtained from the Ethical Review Committee of Debre Markos University, College of Medicine and Health Sciences institutional research review committee (Ref. No/ HSR/R/C/Ser/215/ 11/ 15). As the study was conducted through reviewing medical records of the clients/patients, the individual patients were not subject to harm, and the official letter of co-operation for studied hospitals’ was taken from Debre Markos University. Permission was taken from each study hospital managers and medical ward unit leaders. According to the research review committee of College of Health Sciences, written consent was not required as confidentiality and anonymity were strictly maintained. To keep confidentiality, name and other identifiers of patients and health care professionals were not recorded on the data extraction format. Confidentiality was maintained through anonymity and privacy measures were taken to preserve the right of the participants throughout the research work including publication. This study was conducted in accordance with the Declaration of Helsinki.

## Results

### Socio demographic characteristics of study participants

A total of 680 pregnant women ANC service hospital records were analyzed. About 530 (77.9%) of the study participants were married. About 51.47% (350) of respondents were in the age range of 25–34 (Table 1).

**Table 1:** Socio-demographic characteristics of pregnant women in the West Gojjam Public Hospitals, 2023 (n = 680).

Variables	Category	Frequency (%)
Age	15 −24	210 (30.88)
	25-34	350 (51.47)
	35 or more	120 (17.65)
Marital status	Single	76 (11.20)
	Married	530 (77.90)
	Divorced	74 (10.90)
Residency	Rural	576 (84.70)
	Urban	104 (15.30)
Distance from HF	Less than/equal to 5 km	96 (14.10)
	Greater than 5 km	584 (85.80)

### Obstetrics and gynecological characteristics

About three hundred eighty-six study participants (56.8%) were multiparous and one hundred twenty-one (17.8%) study participants were grand multiparous. Nearly two third of women 456 (67.1%) had wanted pregnancy status. Normal vaginal delivery was the common mode of delivery for 400 (58.8%) followed by assisted delivery for 120 (17.6%). About Sixty-three women (9.26%) had pregnancy-induced hypertension, followed by antepartum hemorrhage in 42 (6.2%), which were the obstetric complications during pregnancy (Table 2).

**Table 2:** Obstetrical/gynecological characteristics of pregnant women in the West Gojjam Public Hospitals, 2023 (n = 680).

Variables	Category	Percentage (%)
Gravidity	Permi-gravida	88 (12.90)
	Multi-gravida	592 (87.10)
Parity	Permi-para	85 (12.50)
	Null Para	88 (12.90)
	Multipara	386 (56.80)
	Grand multipara	121 (17.80)
Status of pregnancy	Unwanted	224 (32.90)
	Wanted	456 (67.10)
Previous mode of delivery	Spontaneous vaginal delivery	400 (58.80)
	Instrumental	120 (17.60)
	C/Section	80 (11.80)
Previous obstetric complication	Yes	131 (19.30)
	No	549 (80.70)
Previous gynecological complication	Yes	146 (21.50)
	No	534 (78.50)
Previous obstetrics complications	Premature rupture of membrane	14 (2.05)
	Pregnancy induced hypertension	63 (9.26)
	Antepartum hemorrhage	42 (6.20)
	Uterine rupture	12 (1.76)

### Services delivered to pregnant women during ANC Visit in public Hospitals

Of the critical ANC components, 95.6% of women were counseled about the danger signs and symptoms occurred during pregnancy by their care givers at each visit of their antenatal care (Table 3).

**Table 3:** Antenatal care services of pregnant women in West Gojjam Public Hospital, 2023 (n = 680).

Components of ANC services	Frequency (%)
Blood pressure measured at each visit	200 (29.40)
Weight taken at each visit	264 (38.80)
Blood sample taken at least two visits	72 (10.60)
Urine sample taken at first visit	416 (61.20)
Provider discussed dangers sign and symptoms at each visit	656 (95.60)
Symphysis-fundal height measured at each visit	264 (38.80)
Provider counsel about healthy diet at each visit	656 (95.60)
Antenatal ultrasound at least one visit	480 (70.60)

**Fig 1:**
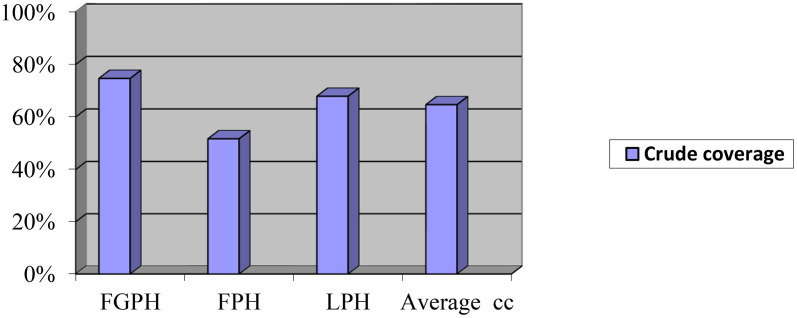
ANC4 attendants of pregnant women in the West Gojjam Public Hospital, 2023; (n = 680). FGPH: Finote Selam General hospital, FPH: Feresbet Primary Hospital, LPH: Liben Primary hospital.

Among women who attended their ANC services in Finoteselam General Hospital (74.7%), Feresbet Primary hospital (51.7%), and Liben primary hospital (67.9%), had received at least four ANC visits, even though when timing and scheduling were not considered (Fig 1).

### Coverage of antenatal care services

Effective coverage for ANC was 25.9% (95% CI, 22.6: 29.4). About 70.6% of mothers were diagnosed using ultrasound, 88.2% of mothers screened for anemia and only 25.9% of mothers were screened for gestational diabetes mellitus at least once during their ANC visit (Table 4).

**Table 4:** ANC Services of pregnant women in the West Gojjam Public Hospital, 2023; (n = 680).

ANC intervention	Received WHO recommended services at least once	Received all WHO recommended services at each visit
Screening for hypertension	640 (94.1%)	200 (29.4%)
SFH measurement	680 (100%)	264 (38.8%)
Screening for anemia	600 (88.2%)	72 (10.6%)
Antenatal ultrasound	480 (70.6%)	56 (8.2%)
Screening for gestational DM	176 (25.9%)	56 (8.2%)
Screening for asymptomatic bacteriuria	416 (61.2%)	416 (61.2%)
Screening for Rh type	544 (80%)	544 (80%)
Screening for tetanus immunization status	600 (88.2%)	600 (88.2%)
Screen for iron/folic supplementation	504 (74.1%)	504 (74.1%)
ANC4+	264 (38.8%)	56 (8.2%)
Effective coverage	176 (25.9%)	56 (8.2%)

### Factors associated with effective coverage of ANC services

In the bi-variable logistic regression analysis, age of respondents, residence, distance from health facilities, marital status, parity, status of pregnancy, previous obstetric complications and previous gynecological complication were identified as a candidate variable for multi variable logistic regression at p-value <0.25. In multi-variable logistic regression analysis age, pregnancy status being wanted pregnancy, presence of previous obstetric complication and presence of previous gynecological complications remained statistically significantly associated with effective coverage of ANC services (Table 5).

**Table 5:** Bi-variable and multivariable logistic regression of effective coverage of ANC among pregnant women in West Gojjam Public Hospitals, 2023; (n = 680).

Variables	Category	Effective coverage		COR (95% CI)	AOR (95% CI)
		Yes	No		
Age	15-24	74	136	1(ref.)	1 (ref.)
	25–34	54	296	0.34 (0.22-0.50)	0.32 (0.20-0.50)
	35 or more	48	72	1.23 (0.77-1.95)	1.55 (0.90-2.67)
Marital status	Single	6	10	1(ref.)	1(ref.)
	Married	143	387	4.31(1.83-10.14)	0.36 (0.12- 1.08)
	Divorced	27	47	6.70 (2.57-17.48)	1.03 (0.57-1.86)
Residency	Rural	160	416	1(ref.)	1(ref.)
	Urban	16	88	0.47(0.27-0.83)	1.43 (0.76-2.70)
Parity	Null Para	32	56	1(ref.)	1(ref.)
	Primi-para	25	60	0.73 (0.69-1.38)	1.15 (0.43- 3.03)
	Multipara	87	299	0.51 (0.31-0.84)	0.54 (0.22- 1.34)
	Grand multipara	32	89	0.63 (0.35-1.14)	0.74 (0.34-1.57)
Status of pregnancy	Unwanted	48	176	1(ref.)	1(refer.)
	Wanted	128	328	0.70 (0.48-1.02)	1.65 (1.08-2.55)
Previous Obstetric complication	Yes	56	49	0.23 (0.15-0.35)	6.27 (3.83-10.28)
	No	120	455	1(ref.)	1(ref.)
Previous gynecological complications	Yes	151	393	0.59 (0.36-0.94)	2.08 (1.16-3.77)
	No	25	111	1(ref.)	1(refer.)

*Significantly associated at p-value <0.05, multivariate analysis.

ref. = reference.

Accordingly, the odds of effective coverage of ANC among mothers with age group of 25–34 years was 68% lower than that of mothers with age group of 15–24years (AOR: 0.32; 95% CI: 0.20, 0.50).

The odds of effective coverage of ANC among mothers with wanted pregnancy were 1.65 times higher than their counterparts (AOR: 1.65; 95% CI: 1.08, 2.55).

Likewise, the odds of effective coverage of ANC among mothers who had experienced previous obstetrics complications were 6.27 times higher than their counter parts (AOR: 6.27; 95%CI: 3.83, 10.28).

The odds of effective coverage of ANC among mothers who had encountered previous gynecological complications were 2.08 times higher than those mothers who had no encountered previous gynecological complications (AOR: 2.08; 95%CI: 1.16,3.77) (Table 5).

## Discussion

The main purpose of this study was to assess the effective coverage of ANC service and associated factors among women attended ANC follow up in the West Gojjam Public hospitals. The overall effective coverage of ANC services was 25.9% (95% CI: 22.6, 29.4). The result implies that in the study population, only about one in four pregnant women received antenatal care (ANC) services that met the full standard of both crude coverage and quality of care. This finding was higher than study conducted in Ethiopia (22%), [23]. This difference might be due to study setting; the former study was conducted nationwide using DHS of 2016 whereas the current was conducted at public hospitals of a single zone. However, this finding is lower than studies conducted in Ethiopia (40%) [29], and Pakistan (35%) [35]. The difference in ANC coverage between the two groups might be because of differences in their social backgrounds, where the study was done, and how the study was conducted.

All pregnant women in our sample had received a SFH (100%) measurement at least once and this result is higher than the findings from studies conducted in Pakistan(51%) [35] and Nepal of ANC content using survey data [14]. A possible justification is that SFH measurement is a simple, low-cost, and non-invasive procedure that is a standard, routine part of every antenatal care visit in public hospitals in this region of Ethiopia. Unlike other more complex interventions that may not require specific equipment, laboratory resources, or specialized training, SFH measurement can be performed by any trained healthcare provider using a basic tape measure. This makes it a highly feasible and consistently delivered component of care, ensuring its near-universal application. The high SFH measurement rate, therefore, likely reflects its integration as a core, mandatory, and easily implementable component of the local antenatal care protocol, which may not be as consistently or universally mandated in the healthcare systems of the comparison countries. This suggests that while other, more resource-intensive interventions might be lacking, this fundamental aspect of maternal health screening is well-established and routinely practiced. In addition to this, this study revealed that the screening for gestational diabetic mellitus during antenatal care was low (25.88%). This indicates a significant gap in the quality and completeness of antenatal care (ANC) services. It suggests that many pregnant women are not being assessed for a condition associated with serious maternal and neonatal complications. The World Health Organization recommends routine testing for hyperglycemia during pregnancy as part of comprehensive ANC. Therefore, the low screening coverage reflects inadequate adherence to guidelines and missed opportunities for early detection and management. Strengthening standardized ANC protocols, ensuring availability of glucose testing supplies, and providing refresher training and supportive supervision for healthcare providers are essential to improve compliance and enhance the quality dimension of effective ANC coverage. This finding was lower than the findings of studies conducted in Pakistan(51%) and Nepal [14]. The possible justification for this difference might be due to study setting and poor adherence to the guidelines.

The finding of this study showed that more than two third of pregnant women had received at least once screening tests for hypertension: 94.1%, antenatal ultrasound: 70.6%, and anemia screening: 88.2%. These results were comparable to the findings of a study conducted in West Bank (hypertension (98%); fetal growth abnormalities (66%); anemia (93%), and antenatal ultrasound (74%)) [24].

As a result, the odds of effective coverage of ANC among mothers with age group of 25–34 years were lower than that of mothers with age group of 15–24years. This finding is contralateral to finding of a study in Nepal [14]. The possible justification might be due to mothers with prior positive pregnancy experiences might perceive subsequent pregnancies as less risky and thus feel less compelled to seek early or frequent ANC. They might rely on their own experience or traditional practices [36]. Women who had multiparty have higher chance of having good effective coverage of ANC service than their counterparts. This finding is supported by a study conducted in Nepal [14] but diverges to the studies conducted in West bank [24]. This difference could be attributed to greater engagement in follow-up and heightened awareness of ANC services within multiparty women.

This study showed that women who had previous obstetrics complication were 6.27 times more likely to have good effective coverage of ANC service than the counterparts. This might be due to mothers who had encountered previous complications understanding the impact of not attending antenatal care. Presence of previous gynecological complication also was 2.08 times more likely to have good effective coverage of ANC service than participants who had no previous complications. The possible reason might be that women who had previous obstetrics and gynecological complications had more ANC follow-up to avert the current obstetrics and gynecological complications. This implies that health systems should ensure that standardized ANC protocols are applied consistently to all pregnant women, regardless of prior history. Messaging and counseling strategies should emphasize that every pregnancy carries potential risks and requires full adherence to recommended ANC content. Additionally, monitoring systems should track not only high-risk follow-up but also completeness of care among low-risk women to prevent inequities in service delivery. This approach would promote equitable, preventive, and comprehensive maternal healthcare.

Moreover, women who had wanted pregnancy history were 1.65 times more likely to have good effective coverage of ANC service than women who had unwanted pregnancy. This might be that women who had wanted pregnancy had more positive perceptions about ANC follow-up than their counterpart.

### Limitation of this study

Crucial variables (income, family size, level of education…) for a comprehensive assessment of effective antenatal coverage were not consistently documented in patient charts, potentially leading to bias in the prevalence estimates. This study tried to assess EC using four or more antenatal care visits and WHO recommended services at least once and this might lead to overestimate of the result. We had no control over the original data collection process, which may have introduced biases or errors that are difficult to identify and account for. Since this study was cross-sectional, it is impossible to conclude that any observed associations directly caused variations in effective antenatal coverage. Therefore, it is better to conduct this study using cohort study design or using primary data for the future to get more valid information.

## Conclusion

This study revealed that one out of four mothers received effective coverage of antenatal care services in the study area. This indicates substantial gaps between the care pregnant women are receiving and the care they need to ensure a healthy pregnancy and birth. Age, wanted pregnancy, presence of previous obstetric complication and presence of previous gynecological complication were significant predictors of effective coverage of ANC service. Therefore, Policymakers should integrate effective coverage indicators into routine monitoring systems and reinforce adherence to national ANC guidelines to guarantee that all pregnant women regardless of age, pregnancy intention, or prior complication history receive the full recommended package of services. Health managers should therefore strengthen standardized ANC checklists, supportive supervision, and regular clinical audits to ensure consistent delivery of essential interventions. In addition, tailored counseling strategies should address differences in risk perception across age groups and provide additional support for women with unintended pregnancies to enhance engagement and continuity of care. Collectively, these actions would promote equitable, preventive, and quality-focused maternal health services in the study setting to improve the number of women receiving effective ANC care.

## Supporting information

S1 File Data set. (RAR)

## Abbreviation:

ANC: Antenatal Care
ANC 4 +: Women who attend four or more ANC Visits
BG: Blood Group
CC: Crude Coverage
DHS: Demographic and Health Survey
EC: Effective Coverage
FANC: focused antenatal care
FP: Family Planning
FMOH: Federal Ministry of Health
GDM: Gestational Diabetic Mellitus
HCT: Hematocrit
LNMP: Last Normal Menstrual Period
LMIC: Low and middle-income countries
MMR: Maternal Mortality Rate
MCH: Maternal and Child Health
PNC: Post Natal Care
QOL: Quality of Life
RH: Rhesus factor
SFH: Symphysis-fundal height
TT: Tetanus Toxoid
U/A: Urine analysis
VDRL: Veneral Diseases Research Laboratory
WHO: World Health Organization
