# Supplementary material for: Effective coverage and associated factors of antenatal care service among women who attended antenatal care at West Gojjam Zone Public Hospitals, Northwest Ethiopia
Source: PLoS One. 2026 Apr 21;21(4):e0347341. doi: 10.1371/journal.pone.0347341 (PMC13098960; doi:10.1371/journal.pone.0347341)
Supplement: Attachment — Submitted filename: Point by point response letter.docx [file pone.0347341.s002.docx]

**Point by point response letter**

**Title: Effective Coverage and Associated Factors of Antenatal Care Service among Women Who Attended Antenatal Care at West Gojjam Zone Public Hospitals, Northwest Ethiopia**

Reviewer's Responses to Questions

**Comments to authors**
1. Is the manuscript technically sound, and do the data support the conclusions?
The manuscript must describe a technically sound piece of scientific research with data that supports the conclusions. Experiments must have been conducted rigorously, with appropriate controls, replication, and sample sizes. The conclusions must be drawn appropriately based on the data presented.

Reviewer #1: No

**Response: Thank you for your comments and we have tried to considered it in the revised manuscript.**

Reviewer #2: Yes

**Response: thank you**

2. Has the statistical analysis been performed appropriately and rigorously?

Reviewer #1: No

**Response: Thank you for your comments and we have tried to considered it in the revised manuscript.**

Reviewer #2: Yes

**Response: Thank you**

3. Have the authors made all data underlying the findings in their manuscript fully available?
The [PLOS Data policy](http://track.editorialmanager.com/CL0/http:%2F%2Fwww.plosone.org%2Fstatic%2Fpolicies.action%23sharing/1/010f0194ffbcbaf8-c9d6e1a0-ac85-4da8-b02c-5317c5d076a0-000000/K5OpjnedO7ERbdZW67QE-mAx8SfRi80d5p5QnPmYkjo=198) requires authors to make all data underlying the findings described in their manuscript fully available without restriction, with rare exception (please refer to the Data Availability Statement in the manuscript PDF file). The data should be provided as part of the manuscript or its supporting information, or deposited to a public repository. For example, in addition to summary statistics, the data points behind means, medians and variance measures should be available. If there are restrictions on publicly sharing data—e.g. participant privacy or use of data from a third party—those must be specified.

Reviewer #1: Yes

Reviewer #2: Yes

**Response: thank you**

4. Is the manuscript presented in an intelligible fashion and written in standard English?

PLOS ONE does not copyedit accepted manuscripts, so the language in submitted articles must be clear, correct, and unambiguous. Any typographical or grammatical errors should be corrected at revision, so please note any specific errors here.

Reviewer #1: Yes

Reviewer #2: Yes

**Response: thank you**

5. Review Comments to the Author
Please use the space provided to explain your answers to the questions above. You may also include additional comments for the author, including concerns about dual publication, research ethics, or publication ethics. (Please upload your review as an attachment if it exceeds 20,000 characters)

Reviewer #1: 1. A revision of Abstract is mandatory, because of too many words and irrelevant result.

**Response:** **We found this concern very important for the betterment of our manuscript and it is considered in the revised manuscript**.
2. Since revision of result is needed, it is too difficult to conclude based on the above findings.

**Response: We have reconsidered it in the revised manuscript**
3. Revision of background section is also seems mandatory because, most of the introduction section deems methods section

**Response: We found this concern very crucial and we have corrected it accordingly.**
4. Who were included and excluded in this study?

**Response**: **We have clearly stated in the manuscript, see line 115 to 118 of the revised manuscript.**
5. Some operational definitions need clarification

**Response: We appreciate the concern and we try to make clear. See line 141 to 157 of the revised manuscript.**
6. If the study was retrospective, would the authors revise the title and study design stated above???

**Response**: **This study was not retrospective cohort rather we use one time secondary data of a certain time period which is cross sectional study. Even though the data spans previously, the analysis will likely treat it as a single snapshot of antenatal coverage during that period. We were not tracking changes over time in a longitudinal manner.**

7. How did you assess independent variables from a chart recorded, for instance distance from health facilities??

**Response: Really impressive. In the chart of the patient residence is recorded. After knowing the place where they come, it well known how far it is from the institution and were collected the data in this way. This particular variable (distance from health institutions) and others were filled from the records of the study participants**.
8. I advise to revise the entire document, especially result and discussion section again.

**Response: Important document and we have tried to revise the manuscript as per your recommendations.**

Reviewer #2: Line 83 "one third of one million" is a very unorthodox means of expressing a number. Please convert to the correct number

**Response: We have revised it accordingly**.
In table 2 it is not clear whether the complications refer to a previous or the current pregnancy. Please make this clear.

**Response: we have corrected accordingly. See the revised table**
To the reader when you discuss and present results concerning effective coverage it is confusing because for the various interventions, not all are recommended to be done at each visit. For example, ultrasound is recommended but normally only to be done once, whereas blood pressure ought to be measured at each encounter. I think you need to include a clear description of the various essential interventions together with a description as to how many times it is recommended each are done throughout a pregnancy. This could then be incorporated into Table 4 for greater clarity

**Response:** **We appreciate the reviewer's insights. While these specific issues were not explicitly detailed in the original manuscript, they were carefully accounted for during outcome measurement, as demonstrated in Table 3 and table 4 of the revised version**

The limitations section is incomplete and stops mid-sentence (lines 202-203) There are several more limitations that should be mentioned.

The discussion should be expanded to include some consideration as to the limitations of so called "effective care". Fully effective care includes not simply taking measurements but also acting upon them if the results give cause. For example, taking a blood pressure measurement is not effective unless there is evidence that high blood pressure receives appropriate attention and subsequent action to treat the problem. Whilst you might not have enough data available to measure this it is still a very important point to discuss and I would like you to do so.

**Response: We tried to advance the discussion of this manuscript accordingly. See the discussion section of the revised manuscript.**
