# Supplementary material for: Effective coverage and associated factors of antenatal care service among women who attended antenatal care at West Gojjam Zone Public Hospitals, Northwest Ethiopia
Source: PLoS One. 2026 Apr 21;21(4):e0347341. doi: 10.1371/journal.pone.0347341 (PMC13098960; doi:10.1371/journal.pone.0347341)
Supplement: Attachment — Submitted filename: PONE_Review.docx [file pone.0347341.s003.docx]

**Manuscript title:**

Effective Coverage and Associated Factors of Antenatal Care Service among Women Who Attended Antenatal Care at West Gojjam Zone Public Hospitals, Northwest Ethiopia

**Competing interests:** I have no competing interest.

This manuscript addresses a critically important topic: the effective coverage of antenatal care (ANC) services in a specific region of Ethiopia. The study's focus on "effective coverage," which integrates need, utilization, and quality, is a valuable approach. The findings highlight a concerningly low effective coverage rate and identify several associated factors. While the topic is highly relevant and the study design is appropriate for its stated aim, the manuscript has some methodological and reporting clarity issues that need to be addressed before publication.

The document exhibits several writing issues that should be addressed. A thorough proofread by a native English speaker or a professional editing service would be highly beneficial. Focus specifically on:

- Correcting all "data was" to "data were."
- Ensuring all percentages in the "Results" section have clearly defined and correct denominators.
- Reviewing interpretations in the "Discussion" for logical consistency with the findings.
- Improving transitions between sentences and paragraphs.
- Adding the crucial missing limitations (selection bias from population definition, short study period).
- Refining precision in word choice and sentence structure throughout.

**Point by point comments**

**Abstract:**

- Reconsider the wording "Despite, there is limited information." It's a bit awkward. "Despite this, limited information exists..." or "However, information is limited..." would be smoother.
- **Study Period:** "from 30 June 2023 to 30 July 2023" is a very short period (one month). While the total sample size (680) is good, it's important to confirm this timeframe. If the study was conducted over such a brief period, consider if this has any implications for the representativeness of your sample.
- **Clarity of Factors:** For categorical variables like "Age 15 to 35," "being married," "divorced," and "multipara," please clarify what the **reference category** was for the AORs. This is essential for interpreting the reported associations. For example, was "Age 15 to 35" compared to "Age <15" or "Age >35"? Was "married" compared to "single"?

Introduction:

- Line 67-70: "It is estimated that every day in 2020, almost 800 women died, maternal death occurred almost every two minute and preventable causes related to pregnancy and childbirth result in the deaths of approximately 330,000 women worldwide every year."
- Please check the consistency of these numbers. "Almost 800 women died every day" (800 x 365 days = 292,000 annually) and "almost every two minute" (which is 30 deaths per hour or 720 per day, equating to ~262,800 annually) seem to contradict the "approximately 330,000 women worldwide every year" statistic. While all are high, it's best to present a single, consistent figure or range with a clear citation for the most recent estimates (e.g., from WHO/UNFPA/World Bank reports) to avoid confusion.
- Line 71-72: "The maternal mortality rate in Ethiopia is high which is, 412 deaths per 100,000 live births" I don’t think this is a recent figure; try to refer recent report.

**Study design and settings:**

1. **Line 94: "An institutional-based analytical cross-sectional study design using secondary data was carried out..."**
   - **Comment:** "Institutional-based" is slightly redundant given you immediately specify "in public hospitals." Consider simplifying to "A facility-based analytical cross-sectional study..."
   - **Suggestion:** "A **facility-based** analytical cross-sectional study was conducted, utilizing secondary data from public hospitals in West Gojjam Zone."
2. **Lines 95-96: "...from 30 June 2023 to 30 July 2023."**
   - **Major Comment:** A **one-month study period** for a cross-sectional study, especially one using secondary data, is remarkably short. This raises significant concerns about the representativeness of the sample and the potential for seasonal variations in ANC attendance or "effective coverage" to be missed.
   - **Question for Authors:** Why was such a short duration chosen? Was this sufficient to achieve the required sample size, considering the average monthly ANC attendance at these hospitals? How do you account for potential monthly or seasonal fluctuations in service utilization?
3. **Lines 96-99: "West Gojjam is one of an administrative zone... As of 2019, the zone had a population of 2,106,596..."**
   - **Suggestion:** Ensure consistency in the reference year for population data. If the study was 2023, why is the population data from 2019? Is there more recent data available, or is 2019 the latest available projection? Acknowledge this if it's the latest available.
4. **Lines 103-107: "The average number of ANC4 visits per year at each hospital was as follows: Finote Selam General Hospital (1,197)... Dembecha Primary Hospital (2)."**
   - **Major Comment:** The figure for **Dembecha Primary Hospital (2 ANC4 visits per year)** is an extreme outlier and highly problematic. This number is incredibly low for a primary hospital providing ANC services.

**Population:**

- There are gross grammatical errors in defining your population please try to revise it.
- Redundancy: your study population and inclusion criteria are a clear repetition.
- Potential for Selection Bias: By focusing solely on women who completed at least four ANC visits within the study hospitals and excluding those who didn’t, your study population might be healthier, more compliant, or face fewer barriers to care than the general population of pregnant women. Please acknowledge this potential selection bias as a limitation

**Sample size determination and sampling procedures**

- The formula should explicitly show how the design effect and non-response rate are incorporated.
- The calculated sample size is approximately 688, not 680 based on your assumption, please justify
- Line 126: "The study participants were chosen using a multi-stage simple random sampling technique." This phrasing is slightly contradictory. It's a "multi-stage sampling technique," and then you specify the type of random sampling used at each stage.
- How did you ensure that there were enough eligible women (those completing ANC4) within that specific one-month period in the selected hospitals to draw a sample of 688?
- What if the ANC4 completers in the 3 selected hospitals during June 30 - July 30, 2023, was less than 688
- Did you checked the adequacy of the calculated sample size for your second objective? Factors associated with EC? You need to show that.

**Measurements and Variables**

- What does "Status of pregnancy" and “Abortion” refer? Clarify
- "investigation and infrastructure related variables (presence of ultrasound, presence of baseline investigation for pregnant women, presence of adequate trained human power, presence of adequate equipment and supplies at ANC unit)"
  - these are facility-level variables, not typically found in individual client charts.
  - How these variables were assessed, how were they operationalized and collected? this needs to be explained in the "Data Collection Tools and Procedures" section.

**Data collection tools and procedures**

- From where did you adapt the questionnaire? Was it based on a WHO checklist template or another established survey tool? Briefly mention.
- Why do you need to translate the questionnaire? Were the medical records of the clients written in Amharic or the data collectors didn’t understand English?
- This part is very brief and lacks critical details needed for a PLOS ONE methods section.

**Data management and analysis**

- How do you calculate EC? Briefly explain the analysis.
- Strict Separation of Methods and Results: remove the Hosmer-Lemeshow P-value and interpretation from the methods section.
- Grammar and Phrasing: Pay attention to subject-verb agreement ("data was" vs. "data were") and awkward phrasing to enhance professionalism and readability.
- Detail for Data Cleaning: While not a major flaw, PLOS ONE encourages authors to briefly mention specific steps taken during data cleaning (e.g., how missing data were handled, checks for outliers). This adds to transparency.

**Ethical considerations**

- Was individual informed consent obtained from each patient whose data was extracted? If not, was the requirement for informed consent **waived** by the ethics committee? If waived, what was the justification for this waiver (e.g., de-identified data, minimal risk, retrospective nature, impracticability of obtaining consent)?

**Results**

Lines 202-203: "Almost half of women 63(48.18%) had pregnancy induced hypertension followed by APH 42(32.1%) from obstetric complication in pregnancy (Table 2)."

63 out of your total sample of 680 is 9.26%, not 48.18%.

Similarly, 42 out of 680 is 6.18%, not 32.1%.

Lines 213-216: "Among women who attended their ANC services in Finoteselam General Hospital (74.7%), Feresbet Primary hospital (51.7%), and Liben primary hospital (67.9%), had received at least four ANC visits, even when timing and scheduling were not taken into account (Figure 1)."

**Major Issue:** Contradiction with Study Population. This sentence directly contradicts your "Population" section, which stated that your study population was defined as pregnant women who attended and completed at least four ANC visits.

**Discussion**

Lines 274-278: "The odds of effective coverage of ANC among mothers with age group of 25-34 years were 68% lower than that of mothers with age group of 15-24years... This could be that this age group is active reproductive group level and had more awareness about the importance of ANC follow-up."

**Major Logical Inconsistency**: If the 25-34 age group is "more aware" and "active," logically one would expect higher (not lower) odds of effective coverage. Your explanation contradicts your finding.

**Limitations**

Line 297: "Certain variables crucial for a comprehensive assessment…

Consider giving one or two specific examples of such variables

Acknowledge other limitations of your study such as:

Selection Bias due to Study Population Definition

Short Data Collection Period

Facility-Level Variables collected from Client Charts?

**Your article needs a thorough revision and lots of edition. Please go through the whole document and edit grammar and language, spelling, and punctuation errors. Addressing these points will significantly improve the manuscript's overall quality and readability for publication.**
