# Supplementary material for: Effective coverage and associated factors of antenatal care service among women who attended antenatal care at West Gojjam Zone Public Hospitals, Northwest Ethiopia
Source: PLoS One. 2026 Apr 21;21(4):e0347341. doi: 10.1371/journal.pone.0347341 (PMC13098960; doi:10.1371/journal.pone.0347341)
Supplement: Attachment — Submitted filename: Point by point response 2.docx [file pone.0347341.s004.docx]

**Point by point response letter**

**Title: Effective Coverage and Associated Factors of Antenatal Care Service among Women Who Attended Antenatal Care at West Gojjam Zone Public Hospitals, Northwest Ethiopia**

**Reviewer #3: Competing interests:** I have no competing interest.

This manuscript addresses a critically important topic: the effective coverage of antenatal care (ANC) services in a specific region of Ethiopia. The study's focus on "effective coverage," which integrates need, utilization, and quality, is a valuable approach. The findings highlight a concerningly low effective coverage rate and identify several associated factors. While the topic is highly relevant and the study design is appropriate for its stated aim, the manuscript has some methodological and reporting clarity issues that need to be addressed before publication.

The document exhibits several writing issues that should be addressed. A thorough proofread by a native English speaker or a professional editing service would be highly beneficial. Focus specifically on:

- Correcting all "data was" to "data were."

**Response: Thank you. It is corrected in the entire document.**

- Ensuring all percentages in the "Results" section have clearly defined and correct denominators.

**Response: We wonder about this insight and corrected it in the revised manuscript.**

- Reviewing interpretations in the "Discussion" for logical consistency with the findings.

**Response:** **Thank you. We found this comment very interesting and corrected accordingly.**

- Improving transitions between sentences and paragraphs.

**Response: corrected accordingly.**

- Adding the crucial missing limitations (selection bias from population definition, short study period).

**Response: We want to forward our great appreciation for the reviewer regarding these issues. The way we write the study population and study period was quite different from the actual study work while manuscript preparation. See the revised manuscript.**

- Refining precision in word choice and sentence structure throughout.

**Response: Corrected accordingly.**

**Point by point comments**

**Abstract:**

- Reconsider the wording "Despite, there is limited information." It's a bit awkward. "Despite this, limited information exists..." or "However, information is limited..." would be smoother.

**Response: thank you. Corrected accordingly. See the revised manuscript**

- **Period:** "from 30 June 2023 to 30 July 2023" is a very short period (one month). While the total sample size (680) is good, it's important to confirm this timeframe. If the study was conducted over such a brief period, consider if this has any implications for the representativeness of your sample.

**Response: Thank You. The above period was the data extraction period while the exact study period was from September 01, 2021, to August 30, 2022. See the revised manuscript**

- **Clarity of Factors:** For categorical variables like "Age 15 to 35," "being married," "divorced," and "multipara," please clarify what the **reference category** was for the AORs. This is essential for interpreting the reported associations. For example, was "Age 15 to 35" compared to "Age <15" or "Age >35"? Was "married" compared to "single"?

**Response: See table 6 of the revised manuscript. We state there.**

Introduction:

- Line 67-70: "It is estimated that every day in 2020, almost 800 women died, maternal death occurred almost every two minute and preventable causes related to pregnancy and childbirth result in the deaths of approximately 330,000 women worldwide every year."
- Please check the consistency of these numbers. "Almost 800 women died every day" (800 x 365 days = 292,000 annually) and "almost every two minute" (which is 30 deaths per hour or 720 per day, equating to ~262,800 annually) seem to contradict the "approximately 330,000 women worldwide every year" statistic. While all are high, it's best to present a single, consistent figure or range with a clear citation for the most recent estimates (e.g., from WHO/UNFPA/World Bank reports) to avoid confusion.

**Response: Thank you, important comment. See line 67 to 72 of the revised manuscript.**

- Line 71-72: "The maternal mortality rate in Ethiopia is high which is, 412 deaths per 100,000 live births” I don’t think this is a recent figure; try to refer recent report.

**Response: very interesting concern. See line 72 to 76 of the revised manuscript.**

**Study design and settings:**

1. **Line 94: "An institutional-based analytical cross-sectional study design using secondary data was carried out..."**
   - **Comment:** "Institutional based" is slightly redundant given you immediately specify "in public hospitals." Consider simplifying "A facility-based analytical cross-sectional study..."
   - **Suggestion:** "A **facility-based** analytical cross-sectional study was conducted, utilizing secondary data from public hospitals in West Gojjam Zone."
   - **Response: Corrected accordingly.**
2. **Lines 95-96: "...from 30 June 2023 to 30 July 2023."**
   - **Major Comment:** A **one-month study period** for a cross-sectional study, especially one using secondary data, is remarkably short. This raises significant concerns about the representativeness of the sample and the potential for seasonal variations in ANC attendance or "effective coverage" to be missed.

**Response: The study period was from September 01, 2021, to August 30, 2022. See the revised manuscript study period.**

- **Question for Authors:** Why was such a short duration chosen? Was this sufficient to achieve the required sample size, considering the average monthly ANC attendance at these hospitals? How do you account for potential monthly or seasonal fluctuations in service utilization?
- **Response: The study period was from September 01, 2021, to August 30, 2022. See the revised manuscript study period**

3. **Lines 96-99: "West Gojjam is one of an administrative zone... As of 2019, the zone had a population of 2,106,596..."**
   - **Suggestion:** Ensure consistency in the reference year for population data. If the study was 2023, why is the population data from 2019? Is there more recent data available, or is 2019 the latest available projection? Acknowledge this if it's the latest available.
   - **Response:** **Thank you! corrected accordingly.** **See the revised manuscript.**
4. **Lines 103-107: "The average number of ANC4 visits per year at each hospital was as follows: Finote Selam General Hospital (1,197)... Dembecha Primary Hospital (2)."**
   - **Major Comment:** The figure for **Dembecha Primary Hospital (2 ANC4 visits per year)** is an extreme outlier and highly problematic. This number is incredibly low for a primary hospital providing ANC services.
   - **Response: Thank you very much! it was a typing error. There were 742 women who attended 4^th^ ANC visit at Dembecha Hospital**.

**Population:**

- There are gross grammatical errors in defining your population please try to revise it.
- Redundancy: your study population and inclusion criteria are a clear repetition.
- Potential for Selection Bias: By focusing solely on women who completed at least four ANC visits within the study hospitals and excluding those who didn’t, your study population might be healthier, more compliant, or face fewer barriers to care than the general population of pregnant women. Please acknowledge this potential selection bias as a limitation

**Response: the study population of study was all pregnant women conducting ANC follow-up at the Maternal and Child Health (MCH) units of selected West Gojjam zone public hospitals during the data collection period. But the former study population we wrote was taken from operational definition of Effective coverage due to a typing error while revising the author’s comment. See the revised manuscript**

**Sample size determination and sampling procedures**

- The formula should explicitly show how the design effect and non-response rate are incorporated.
- The calculated sample size is approximately 688, not 680 based on your assumption, please justify

**Response: thank you for your concern. But still the sample size calculation was correct for us. n=**$\boldsymbol{(}\boldsymbol{Za/2)}^{\boldsymbol{2}}\boldsymbol{(P)(1-P)/}\boldsymbol{(d)}^{\boldsymbol{2}}$ **=1.96*1.96(0.22*.78)/(0.004)^2^**

**=3.84*0.22*0.78/0.0016=411.84 *1.5 design effect=617.76 +10% non-response rate (10*618) = 618+62=680 total sample size**

- Line 126: "The study participants were chosen using a multi-stage simple random sampling technique." This phrasing is slightly contradictory. It's a "multi-stage sampling technique," and then you specify the type of random sampling used at each stage.

**Response: we use more than one stage and lastly, we apply simple random sampling to select the study participants medical registration /chart, as per our experience and level of knowledge such king of sampling technique= multi-stage simple random sampling technique.**

- How did you ensure that there were enough eligible women (those completing ANC4) within that specific one-month period in the selected hospitals to draw a sample of 688?
- **Response: same as above response. The study period was too long not one month**.
- What if the ANC4 completers in the 3 selected hospitals during June 30 - July 30, 2023, was less than 688
- **Response: same as above response. The study period was too long, not one month**.
- Did you checked the adequacy of the calculated sample size for your second objective? Factors associated with EC? You need to show that.

**Response: thank you. It was checked, see table one of the revised manuscript.**

**Measurements and Variables**

- What does "Status of pregnancy" and “Abortion” refer? Clarify
- **Response: Thank you. See the revised manuscript, it is elaborated.**
- "Investigation and infrastructure related variables (presence of ultrasound, presence of baseline investigation for pregnant women, presence of adequate trained human power, presence of adequate equipment and supplies at ANC unit)" these are facility-level variables, not typically found in individual client charts.
  - How these variables were assessed, how were they operationalized and collected? this needs to be explained in the "Data Collection Tools and Procedures" section.
- **Response: Thank you very much! We were used as utilization variable by everyone except for the presence of adequate equipment and supplies at ANC unit variables. presence of adequate equipment and supplies at ANC unit variables were not used during the analysis of data.**

**Data collection tools and procedures**

- From where did you adapt the questionnaire? Was it based on a WHO checklist template or another established survey tool? Briefly mention.

**Response: thank you. See the revised manuscript, we tried to clarify it.**

- Why do you need to translate the questionnaire? Were the medical records of the clients written in Amharic or the data collectors didn’t understand English?

**Response: the questionnaire was prepared in English only. See the revised manuscript.**

- This part is very brief and lacks critical details needed for a PLOS ONE methods section.

**Response: Thank you! see the revised manuscript.**

**Data management and analysis**

- How do you calculate EC? Briefly explain the analysis.

**Response: As we elaborate in the operational definition section, we count mothers who had both “ANC4+ ANC visits and getting WHO recommended services”/ total sample size *100**

- Strict Separation of Methods and Results: remove the Hosmer-Lemeshow P-value and interpretation from the methods section.

**Response: We corrected accordingly.**

- Grammar and Phrasing: Pay attention to subject-verb agreement ("data was" vs. "data were") and awkward phrasing to enhance professionalism and readability.

**Response: Really appreciated comment and we correct through out the manuscript document. We also check the grammar of the whole manuscript document.**

- Detail for Data Cleaning: While not a major flaw, PLOS ONE encourages authors to briefly mention specific steps taken during data cleaning (e.g., how missing data were handled, checks for outliers). This adds to transparency.
- **Response: thank you. There was no missing data.**

**Ethical considerations**

- Was individual-informed consent obtained from each patient whose data was extracted? If not, was the requirement for informed consent **waived** by the ethics committee? If waived, what was the justification for this waiver (e.g., de-identified data, minimal risk, retrospective nature, impracticability of obtaining consent)?

**Response: Thank you for your concern. We tried to elaborate more in the revised manuscript. Consent was waived due to its retrospective nature.**

**Results**

Lines 202-203: "Almost half of women 63(48.18%) had pregnancy induced hypertension followed by APH 42(32.1%) from obstetric complication in pregnancy (Table 2)."

63 out of your total sample of 680 is 9.26%, not 48.18%.

Similarly, 42 out of 680 is 6.18%, not 32.1%.

Response: Thank you. We corrected it accordingly.

Lines 213-216: "Among women who attended their ANC services in Finoteselam General Hospital (74.7%), Feresbet Primary hospital (51.7%), and Liben primary hospital (67.9%), had received at least four ANC visits, even when timing and scheduling were not taken into account (Figure 1)."

**Major Issue:** Contradiction with Study Population. This sentence directly contradicts your "Population" section, which stated that your study population was defined as pregnant women who attended and completed at least four ANC visits.

**Response: Thank you. This comment helps us to correct the methodological problems that we made during manuscript preparation in general. We want to acknowledge you again for your invaluable comments.**

**Discussion**

Lines 274-278: "The odds of effective coverage of ANC among mothers with age group of 25-34 years were 68% lower than that of mothers with age group of 15-24years... This could be that this age group is active reproductive group level and had more awareness about the importance of ANC follow-up."

**Major Logical Inconsistency**: If the 25-34 age group is "more aware" and "active," logically one would expect higher (not lower) odds of effective coverage. Your explanation contradicts your finding.

**Response: Thank you, we corrected it accordingly.**

**Limitations**

Line 297: "Certain variables crucial for a comprehensive assessment…

Consider giving one or two specific examples of such variables

Acknowledge other limitations of your study such as:

Selection Bias due to Study Population Definition

Short Data Collection Period

Facility-Level Variables collected from Client Charts?

**Response: Thank you! All the raised issues are interesting and addressed by this study.**

**Your article needs thorough revision and lots of editions. Please go through the whole document and edit grammar and language, spelling, and punctuation errors. Addressing these points will significantly improve the manuscript's overall quality and readability for publication.**

**Response: thank you very much for your interesting comments. We found your whole comments very important to improve our manuscript and we were happy to correct each comments raised. We believe this manuscript is well improved accordingly.**

Reviewer #4: Abstract: There are a few points that would help sharpen the focus of the abstract. For example, in the phrasing “due to ineffective coverage of antenatal care service and other reason” (line 17), “other reasons” should be specified (e.g., poor health infrastructure, socioeconomic barriers) or removed to sharpen the focus.

**Response: We appreciated this comment and corrected.**

The phase (line 19) “Despite, there is limited information on effective coverage of Antenatal care in Ethiopia” is grammatically incorrect and should be revised (such as, “However, there is limited evidence on the effective coverage of antenatal care in Ethiopia”).

**Response: Corrected in the revised manuscript.**

The abstract would also benefit from an overall grammatical and stylistic editing, such as inconsistent capitalization (antenatal and Antenatal – line 18 versus line 20) t improve the overall clarity.
Response: Corrected accordingly in the revised manuscript.
Introduction: The introduction addresses the critical health issue of maternal mortality and introduces “effective coverage” as a key health system performance metric that integrates the concepts of need, utilization, and quality. This is a highly relevant concept, particularly in the context of maternal and antenatal care (ANC) in low-resource settings. It is also important to see continued work in better measurement of quality-related concepts versus numbers-only indicators to truly measure progress.
In general, it may help to strengthen the introduction to reverse the current order of ideas so that the argument cascades logically from the global to specific. The narrative currently starts at the micro-level by explaining ANC’s benefits and then shifts abruptly to global inequities with the EC framework concepts interspersed. The ideas are already there, but a more logical sequence of the global-to-specific would provide a more logical sequence would build a stronger case for the reader. It will also help readers more readily understand the study’s rationale and the added value of examining effective coverage specifically in the Ethiopian context.For example, you could consider opening with the striking global statistics on maternal mortality, highlighting women in low- and middle-income settings face a higher burden than those in high-income settings (lines 81-85). Then moving to the more specific situation in the Ethiopian setting which provides the context for this study (lines 85-90). This can help establish up front the scale and urgency of the problem. From there, narrowing to the focus on ANC as one of the most important evidence-based interventions for detecting and averting life threatening maternal conditions. This shows readers why ANC quality matters in solving this global challenge (lines 48-57; lines 91-97). Once this context has been established, then you could introduce the concept of effective coverage as the lens through which the study investigates not merely if women attended ANC and enough sessions of ANC, but whether or not they received the quality of services needed (starting with line 58 and remaining sections not cited above).
**Response : we really appreciate this insight and we learn more. We corrected accordingly, see the revised manuscript whole introduction part.**
There are also some points where some adjustments to phrasing would be helpful to more clearly convey the concepts. For example, introducing the concept of effective coverage (line 54), while it is conceptually strong, would benefit from clearer phrasing, such as “Effective coverage is a composite indicator of health system performance…” or perhaps other phrasing that would more clearly express this concept and the intended meaning. Related, there are some circular sentence structures. For example, “use refers to the use of services” (line 58) which offers no additional meaning as is.

**Response: we revised the whole introduction based on your suggestion and our experience to made it preferable to readers**.
There are also some confusing or differing definitions. For example, quality is first defined as “the actual health benefit experienced from the service” (lines 58-59), but later operationalized in terms of receiving the recommended content (lines 62-64). Clarifying this conceptual leap would help improve the flow and strengthen the case you are building.

**Response: we revised the whole introduction based on your suggestion and our experience to made it preferable to readers**

The formula (lines 69-70) is a bit hard to read that just appears to be a formatting issue. Ensuring the equation is formatted more clearly would be helpful to improve comprehension.

**Response: we revised the whole introduction based on your suggestion and our experience to made it preferable to readers**

Another point where the phrasing could be improve is the study objective (lines 101-103). While the objective is clearly stated and aligns well with the background, the wording could be more concise and grammatically polished, such as “The study aimed to assess he effective coverage of antenatal care and identify associated factors among women attending ANC at public hospitals in West Gojjam Zone, North west Ethiopia.”
 **Response: Thank you. Very good insight and corrected as per your suggestion.**
It would also strengthen your argument to provide some more evidence of the situation in Ethiopia around lines 87-90 – such as regional disparities, policy interest in quality improvement, and existing studies (or lack thereof the highlight an important knowledge gap that your study helps to address).It would also help strengthen how this study addresses a knowledge gap by providing some examples of prior studies that focused on crude coverage or perception rather than EC and that none examined the specific zone of the study in order to justify the study’s objective more convincingly.

**Response: we revised the whole introduction based on your suggestion and our experience to made it preferable to readers**
There are some areas where citations are needed, such as the maternal death statistics provided in lines 67-70. There are also some grammatical and stylistic errors that detract from the content. Line 73-76 has a run-on sentence that could be adjusted to two sentences: One possible reason for the persistently high maternal mortality rate could be the poor quality of care, which is not reflected in crude coverage measures. Additionally, the poor uptake of essential interventions during routine ANC visits may have contributed to the stagnant or slow rate of reduction in maternal mortality.

**Response: thank you, done.**
In summary, the content is relevant and valuable but needs improved logical flow, clearer definitions, accurate equation formatting, and some careful language editing to convey the message more effectively.

**Response: Thank you. Very good insight and corrected as per your suggestion**
Methods: The methods section provides a clear overview and includes all the required elements of the study design, setting, and sampling approach. However, several issues reduce the clarity and rigor that can be addressed. Tightening the language, clarifying the sampling procedure, and improving the structure for readability and methodological transparency would benefit this section. Also, please check the size and font of the headings. It seems that everything up through sample size is other “Methods” heading and the remaining (data management, data quality, etc.) under the “Measurement and Variables” heading. It is a bit confusing if this was what was intended.

**Response: Thank you, this comment is important and helps us to amend our manuscript accordingly. See the revised manuscript.**
Study design and setting – The setting is well described but is overall detailed regarding geographic distances and hospital names. This could be condensed for focus and readability.

**Response: Thank you, this comment is important and helps us to amend our manuscript accordingly. See the revised manuscript.**

Population – The phrases “the study population of this study” are redundant (lines 124 and 126). It is also unclear whether “women who completed ANC follow-up” (line 127) means they had all four recommended visits (line 125) that is cited before or a different criterion.
**Response: Thank you. This comment helps us to correct the methodological problems that we made during manuscript preparation in general. We want to acknowledge you again for your invaluable comments.**
Sample size determination and sampling procedures – The sample size calculation is methodologically sound but presented with inconsistent formatting and minor typographical errors (such as line 122 “1.5 design effect” lacks spacing). Consider rephrasing the formula and description of what was used to enhance clarity – perhaps start with the formula that was used, justifying a single population proportion formula, and then the assumptions used for the calculation.
**Response: corrected accordingly.**
The multi-stage random sampling approach is appropriate but the description could be clarified. For example, the phrase “multi-stage simple random sampling” (line 138) is confusing as multi-stage sample and simple random sampling are typically distinct. A more precise explanation of each stage would help. It would also be helpful to note how confidentiality and randomness were ensured with the use of the medical registration numbers (lines 140-142).

**Response: thank you, we elaborated it accordingly.**
Measurement and variables – While this section presents the outcome and explanatory variables clearly in general terms, it would also benefit from further clarity, organization, and precision.
**Response: Corrected, thank you.**
The initial sentence outlining the outcome variable (effective coverage of ANC) is appropriate, but the paper would benefit from further clarity in the definition. The text describes this further in lines 152-156 and notes that women with four or more ANC visits who received “WHO recommended interventions at least once” were considered to have achieved effective coverage. However, this definition is vague in its treatment of timing and frequency. It is unclear whether receiving the interventions only once is considered sufficient, even when guidelines may emphasize repeat or time-specific administration. The list of WHO-recommended interventions (lines 157-160) is presented in a run-on sentence and should be formatted as a clear, concise list to aid readability.

**Response: thank you, we tried to make it clear and precise accordingly. See the revised manuscript.**
But the subsequent listing of independent variables is dense, inconsistently phrased, and lacks logical structure (lines 144-151). The grouping is also confusing, with some sociodemographic variables and some infrastructure variables. Clinical and facility-level variables are intermingled without sub-categorization, which makes this section hard to follow. Organizing by thematic groups (individual-level, clinical history, health system/infrastructure) would significantly improve readability and comprehension.

**Response: thank you very much! We really appreciate your insights and corrected as per your suggestion and scientifically**.
There are a number of abbreviations that are not defined - PROM, APH, PIH, PPH (line 147); PID (line 148); SFH (line 157); HCT,VDRL, BG^RH,U/A (lines 161-162); IESO (line 164); and BP (line 166).

**Response: Done**
The language used throughout this section is often vague or awkward. Terms such as “Gynecology related factors” (line 146) and “Investigation and infrastructure related variables” (lines 148-149) lack specificity and are not clearly defined. Additionally, the phrase “presence of adequate trained human power” (line 150) is grammatically incorrect and could be replaced with more natural phrasing such as “availability of adequately trained personnel.”

**Response: Corrected accordingly.**
There are also typographical errors that detract from the professionalism of the text, such as “pervious History of Gynecology Complication,” (line 148) which should read “previous history of gynecological complications.” Such errors can undermine the credibility of the methodological description.

**Response: Thank you, corrected**
Data collection tools and procedure - This section provides basic information on the data collection tools and procedures but lacks sufficient detail and clarity. While it notes that data were collected using an adapted structured questionnaire translated from English to Amharic, it does not specify the source of the questionnaire, the nature of the adaptation, or whether the tool was pretested or validated. Additionally, the description of data collectors and supervisors is brief and imprecise; stating that three diploma and two BSc nurses were recruited is helpful, but it would be more informative to mention any training they received, how data quality was ensured, and how supervision was conducted.

**Response: thank you, corrected accordingly**
Data quality control - The description in this section is quite general and could benefit from greater specificity. For example, it is unclear what specific adjustments were made following the pretest, or how issues of reliability and data entry accuracy were handled. Additionally, a one-day training duration seems insufficient for complex data collection tasks, especially if the questionnaire included technical or clinical components. More detail on the rationale for that limited training or the quality assurance during data collection and entry to minimize bias or errors would strengthen the credibility of this section.

**Response: the training date was planned to be two days first but due to the nature and the audience level of awareness forced to finish one whole day**.
Data management and analysis - This section needs clearer language and technical precision. There are grammatical errors (e.g., “cheeked” should be “checked” on line 185) and unclear phrasing around model fit. Standard terms like bivariate versus bivariable; multivariate versus multivariable should be used consistently. While the use of p-values to select variables is appropriate, the rationale and handling of issues like multicollinearity or missing data are not discussed. Overall, the section covers key steps but requires clearer writing and more detail for full transparency.

**Response: Corrected as per your suggestion**
Limitation of this study – There is an incomplete sentence here. This section should be reviewed and ensure the full limitations are discussed. Other factors such as the cross-sectional design, possible issues with record-keeping, lack of timing for interventions may also be important limitations to discuss in this section.
**Response: we corrected in the revised manuscript.**
Overall, while this section contains the essential elements required for transparency, it would benefit greatly from improved structure, clearer language, and more thoughtful integration of supporting references. Some sections are underdeveloped and require further elaboration to demonstrate the reliability and rigor of the data collection and analysis processes.

**Response: thank you, your whole comment in this section really very important and we appreciated all concerns you raised and go thoroughly to improve our manuscript.**
Results: The data presented are relevant, the section would benefit from clearer writing and a more logical flow. Examples of some phrasing and formatting that needs improvement, includes:
• “We analyzed and included in the final analysis…” (line 206) could be simplified for clarity, such as “We analyzed 680 ANC service records”;

**Response: corrected, see the revised manuscript.**
• “almost two third” (line 208) should be “almost two-thirds”; also found in line 214

**Response: corrected, see the revised manuscript.**
• line 212-214 could be rephrased, “More than half the women were multipara (56.8%), followed by grand multipara (17.8%)”

• **Response: corrected, see the revised manuscript.**

the “distance from HF” row is missing a parenthesis after 14.1

**Response: corrected, see the revised manuscript.**
• spacing between numbers and parenthesis should be consistent (e.g., Table 2 – some are presented with a space 85 (12.5) and some without 386(56.8)). This is found in the remaining tables.

**Response: corrected, see the revised manuscript.**
• Data presentation should be consistent – most present 1 digit, but line 216 presents 2 digits (48.18%)

**Response: corrected, see the revised manuscript.**
• Line 217 should read “obstetric complications” or perhaps better rewritten “Among women who experienced obstetric complications during pregnancy, nearly half (48.2%) had pregnancy-induced hypertension, followed by antepartum hemorrhage (32.1%)”

**Response: corrected accordingly, see the revised manuscript.**
• Line 228-229 – select consistent capitalization for Hospital or hospital.

**Response: we used both these words, for title of tables and figures “Hospital” and body of the paragraphs or sentences “hospital.”**
Similar suggestions are found throughout the remaining results section and should continue to check for this consistency in presentation, logical flow, and clarity of presentation.
**Response: corrected accordingly, see the revised manuscript.**
Descriptions of the results presented in the tables could be improved. For example, in the description for Table 1, the text notes that 72.9% were in the age range 21-35 but the table shows groups 15-24, 25-34, and 35 or more. The description should match the way data is presented. If there is a reason for discussing results differently than how it is presented in the table, this should be explained.

**Response: thank you: corrected accordingly.**
The description for Table 4 of very unclear, as is why the CI is presented in text and not the table. Are these univariates among all 680 women who had four or more visits? It is also unclear where the description of “80% of mothers were diagnosed using ultrasound” is coming from (line 237-238).

**Response: thank you, really there was typing error while we checked again. Regarding the confidence interval as we are doing point prevalence to discuss later the 95%CI of the outcome variable should reported here. We feel still as very important report.**
The section around table 5, which presents findings from bi-variable and multivariable logistic regression, lacks clarity, consistency, and precision in language, which makes it a bit hard to follow. The opening sentence is overly long and awkwardly constructed. Phrases like “identified as a candidate variables” (line 246) contain both grammatical and pluralization errors. It would benefit from clearer subject-verb agreement and tighter phrasing. The use of "multiple logistic regression" should be replaced with the standard term "multivariable logistic regression" for accuracy.

**Response: corrected accordingly, see the revised manuscript.**
Additionally, the narrative interpretation of odds ratios is not always intuitive or clearly explained. For example, stating that the "odds were 68% lower" when the AOR is 0.32 may confuse readers unfamiliar with interpreting odds ratios. This could be better framed as "women aged 25–34 had significantly lower odds of effective coverage compared to those aged 15–24."

Response: Here we have reservation regarding your comment. As you know in the measure of association, If AOR is below one the interpretation is like we state in the manuscript. <https://www.researchgate.net/post/How_can_interpret_the_value_of_adjusted_odd_ratio_AOR_equal_to_less_than_1_in_binary_logistic_regression>

There is also some repetition and awkward phrasing. For example, "those mothers who had no encountered previous gynecological complications" (lines 260-261) should be revised for grammatical accuracy ("those who had not experienced..."). Finally, references to “table 5” should ideally be integrated more naturally into the narrative or visually indicated in the formatting, especially when listing statistical results.
**Response: corrected accordingly, see the revised manuscript.**
Overall, the results section communicates important findings but would benefit from clearer language, grammatical revision, and improved statistical interpretation.
**Response: thank you, your whole comment in this section really very important and we appreciated all concerns you raised and go thoroughly to improve our manuscript.**
Discussion: The discussion summarizes key findings but is weakened by problems in structure, logic, and writing quality. There is a lot of repeating of the results but no interpretation of those results is provided.
The opening paragraph contains an incomplete phrase - “The overall effective coverage of ANC services 25.9%” (line 270) and immediately compares results with previous studies without first interpreting their substantive meaning of the result found in this study. Comparisons to earlier Ethiopian and Pakistani studies are offered, yet the explanations (“differences in social backgrounds” or “where the study was done”) are speculative and unsupported by data. Citing concrete contextual factors from these comparative studies (e.g., sampling frame, facility type, survey year) would be more convincing and better ground your argument.
**Response: corrected accordingly, see the revised manuscript.**
Throughout, percentages are repeated without context, interpretation, and discussion. For example, readers are told that SFH measurement was 100% (line 278) and that gestational diabetes screening was 8.2% (line 281). But what this means in the Ethiopian – or larger – context or the implications for maternal outcomes or service quality are not discussed. This fails to help the reader understand the significance of these findings.

**Response: We appreciated, corrected accordingly, see the revised manuscript.**
Interpretations of odds ratios are sometimes inaccurate or poorly framed. For example, describing the adjusted odds ratio of 0.32 for women aged 25–34 as “68% lower” is mathematically correct (line 290), yet no rationale is provided for why younger women achieve higher effective coverage. Speculation about “active reproductive group level” (line 292) lacks clarity and evidence. 
**Response: We appreciated, corrected accordingly, see the revised manuscript.**
Similarly, attributing higher coverage among multiparous women to “more exercise about its follow up” (line 297) is vague and grammatically incorrect. The discussion routinely labels findings as “contralateral” or “contrast,” but it fails to explore potential explanations such as health seeking behavior, facility readiness, or cultural norms. Other grammatically confusing or incorrect sentences can be found throughout as well - (“this could be that this age group is active reproductive group level” (lines 291-292); “women who had multiparty have more exercise about its follow up” (line 293-294).
**Response: We appreciated and corrected accordingly, see the revised manuscript.**
Overall, a more effective approach for the discussion section would be to highlight the most salient associations, relate them to existing literature, and consider programmatic implications. This section also requires clearer writing, stronger linkage between results and interpretation, and evidence based explanations for disparities.
**Response: Thank you, we have gone thorough it. See the revised manuscript.**
Conclusion: The conclusion lacks a strong impact. It requires refinement to offer a more coherent and policy-relevant summary of the study’s implications.

**Response: Done accordingly**.

**Reviewer #5**: Thank you for the opportunity to review your manuscript. This is an important topic and could have a major impact on maternal and perinatal mortality. The study estimated the effective coverage of antenatal care in women who had at least 4 AN appointments. The medical records of 680 women who attended antenatal care from 30 June 2023 to 30 July 2023 in one of 3 randomly selected hospitals out of the 8 in West Gojjam Northwest Ethiopia were reviewed by trained data collectors.
The study aims to address an important issue and provides valuable estimates of the rates of crude and effective coverage. However there are some significant limitations with the statistical analysis.
Abstract - I struggled to match the CIs in lines 37-40 with adjusted odds ratios presented in table 5.
**Response:** **Sorry, We didn’t found the raised issue in our manuscript. We have checked the whole manuscript as well our data set and not found discrepancy**. **See the revised manuscript.**
Main text - Consideration of Selection bias - How well did the study population match the source population in terms of demographics? Did the criteria of requiring 4 ANC affect recruitment? One of the hospitals selected was the larger district hospital - are the women who attend this larger hospital comparable to those attending the 2 smaller local hospitals?

**Response: Thank you very much, the population we were studied are clearly stated in the revised manuscript. There was typing error between the operational definition and the study population during manuscript preparation phase.**

The tables and figure titles should be clearer eg Table 1: shows the demographics of pregnant women in the 3 hospitals in the study, not the region. Rather than referring to the region, these tables and figures should referring to 640 women with ante-natal care in 3 hospitals in the province, during one month June- July 2023
**Response: Corrected as per our title of the research.**
Effective coverage only shown in total and not by hospital, but there were clear differences in crude coverage across the 3 hospitals. I would recommend that hospital should be included in the regression modelling in separate models, as a fixed effect but also in a multilevel model as a random effect, to explore within and between hospital variance. This impact of this and confounding across variables mean the results of the multivariable regression presented are likely unstable. Was the likely confounding across many of the variables assessed? Of particular concern are estimates that have changed direction between the bivariate and multivariate analyses; not all of these variables are appropriate to be included in the same model. eg age is confounded with multiple variables including parity, planned pregnancy and marital status.
**Response: Very interesting issue, but our manuscript was prepared as per our study objectives. We want to assess the overall effective coverage of antenatal care figure in West Gojjam zone not for each hospital.**
The discussion would benefit from expansion and should include reference to selection bias and limitations in the statistical analyses.

**Response: see the revised manuscript. It answers the raised issue.**

**Reviewer #6:** The authors present an interesting research question aimed at quantifying effective antenatal care and they propose to examine multiple variables that may impact care. Unfortunately, the manuscript lacks any sound conclusions, presents inconsistent data, and has numerous typographical and grammar errors making it unsuitable for publication at this time.

The most concerning problem with this manuscript is that the data the authors present is inconsistent throughout, leaving the reader unclear what is truth and how that impacts their research question. For example, in the manuscript’s abstract the authors cite “Parity (multipara) (AOR: 1.74; 95 % CI:0.05- 0.55)” but that data is not repeated or presented anywhere in the paper that follows. The main source of this data is in Table 5, but the numbers in that table are different from what’s stated in the abstract. This happens for multiple of the key variables they explored.

**Response: We have checked our manuscript, and we didn’t found these raised figures in our manuscript. If so, could you please see the revised manuscript AOR reports of table 6 of the revised manuscript? Thank you for your genuine comment**.
Also, many of their AORs and CIs don’t make sense where the AOR does not fall within the 95% CI. This is seen in the “Parity (multipara)” example stated above, the “previous gynecological complication (AOR:3.98; 95% CI; 1.33-3.70)” cited in the abstract, and also in the manuscript’s Results and Discussion sections.

**Response: See table 6 of the revised manuscript. No such figure there.**
Finally, the conclusions reached in this manuscript aren’t actually conclusions, but statements about the data. It’s not meaningful to just state which variables are significantly associated with (in)effective coverage of ANC services, the authors should also explain if these variables positively or negatively impacted coverage. For example, did being married or divorced increase or decrease effective coverage? Both have positive AORs and highly variable CIs.

Response: thank you, we have amended the conclusion section of our manuscript. See the revised manuscript.

**Overall, we found the comments of the reviewers very interesting and gives more lesson and we have improved our manuscript as per the raised suggestions and comments from the reviewers. We were very happy due to getting such invaluable comments.**
