# Supplementary material for: Effective coverage and associated factors of antenatal care service among women who attended antenatal care at West Gojjam Zone Public Hospitals, Northwest Ethiopia
Source: PLoS One. 2026 Apr 21;21(4):e0347341. doi: 10.1371/journal.pone.0347341 (PMC13098960; doi:10.1371/journal.pone.0347341)
Supplement: Attachment — Submitted filename: Point by point response letter 3.docx [file pone.0347341.s005.docx]

**Point by point response letter**

**Title: Effective Coverage and Associated Factors of Antenatal Care Service among Women Who Attended Antenatal Care at West Gojjam Zone Public Hospitals, Northwest Ethiopia**

Reviewer's Responses to Questions

**Comments to the Author**
1. If the authors have adequately addressed your comments raised in a previous round of review and you feel that this manuscript is now acceptable for publication, you may indicate that here to bypass the “Comments to the Author” section, enter your conflict of interest statement in the “Confidential to Editor” section, and submit your "Accept" recommendation.

Reviewer #4: All comments have been addressed

Reviewer #6: (No Response)

**Response:** **Thank you! We have tried to address the remaining comments in the revised manuscript.**

2. Is the manuscript technically sound, and do the data support the conclusions?
The manuscript must describe a technically sound piece of scientific research with data that supports the conclusions. Experiments must have been conducted rigorously, with appropriate controls, replication, and sample sizes. The conclusions must be drawn appropriately based on the data presented.

Reviewer #4: Yes

Reviewer #6: Partly

**Response:** Thank you!

3. Has the statistical analysis been performed appropriately and rigorously?

Reviewer #4: Yes

Reviewer #6: Yes

**Response:** Thank you!

4. Have the authors made all data underlying the findings in their manuscript fully available?

The [PLOS Data policy](http://track.editorialmanager.com/CL0/http:%2F%2Fwww.plosone.org%2Fstatic%2Fpolicies.action%23sharing/1/010f019a2ddc486e-03169616-8064-4a5f-a927-850394d37e63-000000/I_BwClgt-ckLQ3z2jxkOz0AecyY_-P8AtU0xbpQSAgM=235) requires authors to make all data underlying the findings described in their manuscript fully available without restriction, with rare exception (please refer to the Data Availability Statement in the manuscript PDF file). The data should be provided as part of the manuscript or its supporting information, or deposited to a public repository. For example, in addition to summary statistics, the data points behind means, medians and variance measures should be available. If there are restrictions on publicly sharing data—e.g. participant privacy or use of data from a third party—those must be specified.

Reviewer #4: Yes

Reviewer #6: Yes

**Response:** Thank you!

5. Is the manuscript presented in an intelligible fashion and written in standard English?

PLOS ONE does not copyedit accepted manuscripts, so the language in submitted articles must be clear, correct, and unambiguous. Any typographical or grammatical errors should be corrected at revision, so please note any specific errors here.

Reviewer #4: Yes

Reviewer #6: Yes

**Response:** Thank you!

6. Review Comments to the Author

Please use the space provided to explain your answers to the questions above. You may also include additional comments for the author, including concerns about dual publication, research ethics, or publication ethics. (Please upload your review as an attachment if it exceeds 20,000 characters)

Reviewer #4: I appreciate the authors’ attention to responding effectively to the previous issues, resulting in a much-improved version of the manuscript. Despite these advancements, the paper is still hindered by grammatical errors, unclear sentence structures, and inconsistencies that detract from clarity and weaken its overall impact.

**Response:** **we really appreciated the raised comments and as much as possible we have tried a lot to correct the grammatical errors in the revised version of the manuscript**.
The Introduction effectively contextualizes the problem, defines key terms, and highlights the study’s relevance. While it is generally well-structured with a clear research gap and notable improvements in flow, it would benefit from minor polishing to address run-on sentences, redundancy, and enhance conciseness.

**Response: Thank you! It is very important comment and we have corrected the introduction section accordingly.**

The Methods section is logically organized with adequate detail but requires further editing for grammar, formatting, and clarity. Addressing awkward phrasing, run-on sentences, and inconsistent tense usage will improve readability and academic rigor, ensuring the manuscript is publication-ready.
**Response: Thank you! It is very important comment and we have corrected the whole method sections accordingly.**

The Discussion is content-rich, appropriately identifies patterns, explains findings, and addresses limitations. However, it needs revision to improve grammar, sentence structure, and logical flow; eliminate redundancy; clarify contradictory points (notably regarding age versus parity); and strengthen the conclusion with actionable recommendations.

**Response: we really wonder this issue. It is corrected accordingly. See the revised manuscript.**

Overall, the manuscript demonstrates commendable attention to detail and effectively addresses the key issues previously raised. I commend the authors for their work and encourage a thorough final edit to resolve remaining grammatical issues, improve clarity, and ensure consistency for a polished, professional presentation.

**Response: Thank you! It is very important comments and we corrected the revised manuscript accordingly.**

Reviewer #6: The original reviewers note, correctly, that a 1 month study period is extremely short for a cross-sectional study design. The authors responded to this comment noting that the study period was actually 1 year and that detail was updated in the revised manuscript, however the abstract in the revised version clearly states only a 1 month study period from 30 June 2023 to 30 July 2023. Then in the Methods Section the authors note the longer time-period from 01 September 2021 - 30 August 2022, but it's unclear why they classify this as "secondary data" and how the data in the 1-month and 1-year study periods are related. Are all of these results included in the results section or only some? It's very unclear and should be updated.

**Response: we really appreciated the concern. This study is a facility based cross- sectional study. The data were collected from pregnant women ANC follow up chart (those pregnant women who had ANC follow up at study area public hospitals from 01 September 2021 - 30 August 2022). Even if the study period was a year i.e. from 01 September 2021 - 30 August 2022, we extracted the data from pregnant mothers ANC follow up chart from 30 June 2023 to 30 July 2023 (a month). Therefore, the study period and actual data extraction/collection period were different, i.e. why we put the two time period here. This study uses secondary data because the data were extracted from clients chart, not directly collected from study participants.**

The overall goal/conclusion of this study was to measure the effective ANC coverage for this population and the authors clearly define effective ANC coverage as "attended four or more antenatal care (ANC) visits, and they received all the WHO recommended interventions at least once during". Given this definition, it does not make sense that the authors conclude that the effective ANC coverage is 25.9%. How is this possible if Table 5 shows that only 8.2% of women received "Screening for gestational diabetes mellitus" which is one of the noted/required WHO interventions? Given this data and how the authors define effective coverage, wouldn't that mean only 8.2% met the standard for effective coverage? The breakdown in services and what is/isn't provided to pregnant individuals in this population is very interesting, but I think the data interpretation doesn't match what the authors themselves laid out as qualifying as effective coverage.

**Response: Really, pleased comment. We were confused while we see this comment and we have checked our data set from the beginning to the end and we found that 8.2 % were “Screening for gestational diabetes mellitus” at each visit as we put in the table 5 of the revised manuscript ( line 255). But the pregnant women who had received “Screening for gestational diabetes mellitus at least once was 25.88%, see the revised manuscript” and this inconvenience was occurred during manuscript preparation. As you know, to conduct statistical analysis determining the outcome variable status is first above all. We were done that considering the above figure (25.88%) for “screening for gestational diabetes mellitus’’ at least once. Lastly, we appreciated a lot in this regard since it is very crucial for the thoroughness of our manuscript.**

**Generally, we feel that your valuable comments and insights make our manuscript well improved and we are too happy due to getting such very important inputs.**
