# Supplementary material for: Effective coverage and associated factors of antenatal care service among women who attended antenatal care at West Gojjam Zone Public Hospitals, Northwest Ethiopia
Source: PLoS One. 2026 Apr 21;21(4):e0347341. doi: 10.1371/journal.pone.0347341 (PMC13098960; doi:10.1371/journal.pone.0347341)
Supplement: Attachment — Submitted filename: point by point response.docx [file pone.0347341.s006.docx]

**Point by point response**

Title: Effective Coverage and Associated Factors of Antenatal Care Service among Women Who Attended Antenatal Care at West Gojjam Zone Public Hospitals, Northwest Ethiopia

**Comments to the Author**
1. If the authors have adequately addressed your comments raised in a previous round of review and you feel that this manuscript is now acceptable for publication, you may indicate that here to bypass the “Comments to the Author” section, enter your conflict of interest statement in the “Confidential to Editor” section, and submit your "Accept" recommendation.

Reviewer #7: All comments have been addressed

Reviewer #8: All comments have been addressed

2. Is the manuscript technically sound, and do the data support the conclusions?
The manuscript must describe a technically sound piece of scientific research with data that supports the conclusions. Experiments must have been conducted rigorously, with appropriate controls, replication, and sample sizes. The conclusions must be drawn appropriately based on the data presented.

Reviewer #7: Yes

Reviewer #8: Yes

3. Has the statistical analysis been performed appropriately and rigorously?

Reviewer #7: Yes

Reviewer #8: Yes

4. Have the authors made all data underlying the findings in their manuscript fully available?
The [PLOS Data policy](http://track.editorialmanager.com/CL0/http:%2F%2Fwww.plosone.org%2Fstatic%2Fpolicies.action%23sharing/1/010f019c888b85ed-85d5dc17-eadb-46c2-ba75-34b52993acd9-000000/j235GL2E17hGxHlSxueRUmo1UqFMVbsvhqeOCfRSH60=251) requires authors to make all data underlying the findings described in their manuscript fully available without restriction, with rare exception (please refer to the Data Availability Statement in the manuscript PDF file). The data should be provided as part of the manuscript or its supporting information, or deposited to a public repository. For example, in addition to summary statistics, the data points behind means, medians and variance measures should be available. If there are restrictions on publicly sharing data—e.g. participant privacy or use of data from a third party those must be specified.

Reviewer #7: Yes

Reviewer #8: Yes

5. Is the manuscript presented in an intelligible fashion and written in standard English?
PLOS ONE does not copyedit accepted manuscripts, so the language in submitted articles must be clear, correct, and unambiguous. Any typographical or grammatical errors should be corrected at revision, so please note any specific errors here.

Reviewer #7: Yes

Reviewer #8: Yes

6. Review Comments to the Author
Please use the space provided to explain your answers to the questions above. You may also include additional comments for the author, including concerns about dual publication, research ethics, or publication ethics. (Please upload your review as an attachment if it exceeds 20,000 characters)

Reviewer #7: Reviewer Comments for Manuscript PONE-D-24-45499R3
1. This manuscript contains grammatical errors, awkward phrasing, and inconsistent tense usage that reduce clarity and readability. Several sentences are overly long or redundant, which weakens the logical flow of the argument. I recommend a thorough language edit to: Correct grammar and typographical errors

**Response:** We really appreciate your concern and we have seen thoroughly the whole and document and corrected accordingly. See the revised manuscript.
• Ensure consistent use of past tense in the Methods and Results sections, and present tense in the Introduction and Discussion…(e.g., “effective coverage,” “crude coverage”)

**Response:** It is interesting and we corrected it accordingly.
• Shorten or restructure overly long sentences to improve conciseness and readability. Minor typographical errors and redundancies should be carefully polished before publication.

**Response:** corrected accordingly.
Suggestion: The manuscript would benefit significantly from thorough proofreading by a native English speaker or professional editing service to improve clarity and adherence to academic writing standards.

**Response:** we will be happy if it is done.
2. Abstract
• The abstract is informative but needs refinement for clarity.

**Response:** Thank you. We have tried to correct as per your suggestion.
• The study period is confusing: the abstract mentions June–July 2023 (1 month), while the methods section refers to September 2021–August 2022 (1 year). This inconsistency must be corrected. You have to remove … “using secondary data of mothers who received antenatal care from September 01, 2021, to August 30, 2022”.

**Response:** corrected. See line 103 to 105 of the revised manuscript.

You have also change design into “retrospective cross-sectional studies”

**Response:** really, corrected accordingly.

. Change…”bi variable logistic regression” → “bivariable logistic regression”;

**Response:** corrected.
• The results section of the abstract should align with the operational definition of effective coverage. For example, if screening for gestational diabetes is required, how does the reported 25.9% coverage reconcile with only 8.2% receiving that service?

**Response:** It is very interesting concern and corrected accordingly. See the revised manuscript table 4.
3. Introduction
• The introduction is well contextualized, but some sentences are repetitive and could be streamlined.

**Response:** corrected accordingly
• The rationale for focusing on effective coverage rather than crude coverage is well stated, but the flow could be improved by reducing redundancy.

Response: corrected accordingly
• Consider clarifying the conceptual framework of effective coverage (need, use, quality) more succinctly.

**Response:** corrected accordingly
• Suggestion: Consider adding a sentence on why West Gojjam Zone specifically was chosen—e.g., known gaps in ANC quality, high maternal mortality, or representative setting.

**Response:** we tried to add evidence in this regard. See the revised manuscript.
• Question: The authors state that “none have examined EC of ANC in our region especially in my study setting.” Were there any sub-national or facility-based ANC quality assessments that could be referenced for context?

**Response:** corrected accordingly.
Methods
Study design and period
The description of the study design is somewhat confusing. The manuscript currently states: “A facility-based analytical cross-sectional study design was conducted in West Gojjam public hospitals from June 30 to July 30, 2023, utilizing secondary data from mothers who received antenatal care between September 1, 2021, and August 30, 2022.”

**Response:** corrected accordingly, see line 103 to 105 of the revised manuscript.
• Since the data were extracted retrospectively from ANC charts, the design would be more accurately described as a retrospective cross-sectional study rather than “analytical cross-sectional.”

**Response:** Really, it should be.
• The phrase “utilizing secondary data from mothers who received antenatal care between September 1, 2021, and August 30, 2022” is redundant here, because the study population and source of data are already explained elsewhere.

**Response:** The redundancy is avoided. See the revised manuscript.
• I recommend that the authors simplify this section to report only the study design and data collection period, while clearly noting that secondary data were used. For example: “A facility-based retrospective cross-sectional study was conducted in West Gojjam public hospitals. Data were collected between June 30 and July 30, 2023, from ANC charts covering the period September 1, 2021, to August 30, 2022.”

**Response:** corrected accordingly
Sampling Size Calculation
I appreciate that the authors calculated the sample size for both objectives (coverage and associated factors). However, including the full formula in the manuscript is not necessary, since you have already stated that the single population proportion formula was used.
I recommend simplifying this section by: Clearly stating that the sample size was calculated for both objectives. Indicating that the largest sample size obtained was selected as the final sample size for the study. Removing the redundant formula presentation, while keeping the assumptions (confidence level, margin of error, prevalence, design effect, non-response rate) clearly described. This will make the Methods section more concise and reader friendly, while still transparent about how the sample size was determined.

**Response:** corrected accordingly. See the revised manuscript line 130 to 137.
Study Design and Sampling:
• The multi-stage random sampling is appropriate, but the description of selecting 3 out of 8 hospitals (38%) by “lottery” is vague. Were all hospitals eligible? Was stratification considered?
• Suggestion: Clarify whether the selection was truly random or purposive, and justify the sample size allocation.

**Response:** Thank you for the valuable comments regarding the sampling procedure. We have clarified the sampling approach as follows: All eight public hospitals in West Gojjam Zone were eligible for inclusion. There were no exclusion criteria. Since the hospitals are relatively homogeneous in terms of service structure, level of care (general and primary hospitals), and provision of antenatal care services under the same regional guidelines, stratification was not considered necessary. The selection of three hospitals was conducted using a simple random sampling (lottery) method. The names of all eight hospitals were written on separate pieces of paper, mixed thoroughly, and three were drawn randomly. Therefore, the selection was entirely random and not purposive.
Why were only 3 hospitals selected? Was power considered at the hospital level?

**Response:** The number of hospitals selected was determined primarily based on feasibility, logistics, and available resources and the recommended selection of study site is 20 to 30% and we took 30 % (the maximum) and 8*30%=2.4 which is approximate to 3: still ensuring representativeness of the zone. The primary unit of analysis in this study was the individual mother (not the hospital). The sample size calculation was based on estimating the prevalence of effective ANC coverage at the individual level. Therefore, statistical power was calculated at the participant level rather than the hospital level. The study was not powered to detect differences between hospitals. Why was a design effect of 1.5 applied? Was clustering expected, and if so, at what level? Please justify this choice.

**Response:** A design effect of 1.5 was applied to account for potential clustering at the hospital level, as participants were nested within selected hospitals. Although individual-level simple random sampling was used within each hospital, responses may be correlated due to shared service environment, provider practices, and institutional characteristics. Since prior intra-cluster correlation (ICC) estimates for effective ANC coverage in similar settings were not available, a conservative design effect of 1.5 was used, which is commonly applied in health service research conducted in similar contexts. This adjustment helped to maintain adequate precision despite possible clustering. <https://www.google.com/search?q=DESIGNING+AND+SELECTING+THE+SAMPLE&oq=DESIGNING+AND+SELECTING+THE+SAMPLE&gs_lcrp=EgZjaHJvbWUyBggAEEUYOTIICAEQABgWGB4yBwgCEAAY7wUyCggDEAAYgAQYogQyBwgEEAAY7wUyCggFEAAYgAQYogQyCggGEAAYgAQYogTSAQkxODc0ajBqMTWoAgywAgHxBWtFjwtMWGeG&sourceid=chrome&ie=UTF-8>
Measurement of Effective Coverage:
The definition aligns with WHO recommendations, but the operationalization—“received all the WHO recommended interventions at least once”may overlook frequency and timing of interventions, which is acknowledged as a limitation.

**Response:** Thank you, we have considered it as a limitation in the revised manuscript.
Suggestion: Briefly note how missing data on intervention timing was handled.
How was “adequately trained human power” assessed? Was it based on presence per shift, or per patient load?

Response: it was done per patient load.
Clarify whether data were extracted only from charts or supplemented with interviews.

**Response:** only chart review.
Line 181-182. Avoid----◊remove…”The questionnaire was prepared in English and
182 pretested among 5% (34) of study population before the actual data collection”…this repetition/The author already included in data quality control.

**Response:** done.
Data Analysis:
The use of p<0.25 for inclusion in multivariable analysis is acceptable but should be justified given the sample size.

**Response:** The use of p < 0.25 as a screening criterion for inclusion in the multivariable logistic regression model was guided by methodological recommendations in regression modeling. This relatively liberal threshold is commonly applied at the bivariable stage to avoid excluding potentially important variables that may not show strong crude associations but could become significant after adjustment for confounding. Given our total sample size of **n = 680**, the study had adequate power to accommodate this approach. The number of outcome events was sufficient to maintain an acceptable events-per-variable (EPV) ratio in the final multivariable model. To prevent over fitting, we ensured that: only theoretically plausible variables were considered, the final model satisfied EPV assumptions (≥10 events per predictor variable)and multicollinearity was assessed before model fitting. Therefore, using p < 0.25 at the bivariable level allowed us to reduce the risk of residual confounding while maintaining model stability and statistical validity.
Suggestion: Consider also using variance inflation factors (VIF) to check multicollinearity, especially among obstetric/gynecological variables.

**Response:** multicollinearity was checked before fitting the final model of regression using variance inflation factor and tolerance test and there was no correlation b/n independent factors.
Question: Why were hospital-level factors (e.g., equipment, staffing) not included in the regression model?

**Response:** Hospital-level variables were not included because the study focused on individual-level determinants, only three hospitals were included (insufficient for stable cluster-level modeling), and facilities were structurally similar under the same regional standards. Future research using larger numbers of hospitals and multilevel analysis is recommended.
Results:
Tables are comprehensive but could be better formatted. For example, Table 5 is split across pages Comment on Definition and Results of Effective Coverage
The manuscript defines effective coverage of ANC interventions as: pregnant women who attended four or more ANC visits and received all WHO recommended interventions at least once during their ANC follow up period. However, the results presented raise important concerns: For several interventions, the proportion of women receiving them “at least once” is far below 25.9%. For example, only 8.2% received antenatal ultrasound and 8.2% were screened for gestational diabetes mellitus. If all WHO recommended interventions are required for effective coverage, then the reported 25.9% effective coverage seems inconsistent with these low uptake rates.

**Response**: the operational definition and the result are consistent as per our research work. See table 4 of the revised manuscript. We tried to make it clear.
• The table also mixes “at each visit” and “at least once” indicators, which makes interpretation difficult. For example, gestational diabetes screening is reported as 25.9% “at each visit” but only 8.2% “at least once.” This needs clarification.

**Response:** see table 4 of the revised manuscript. We tried to make it clear. 25. 9% were screened for GDM at least once and 8.2% were screened for GDM at each visit and the Effective coverage was measured ANC4+ and WHO recommended services at least once. Hence, there is no inconsistence result here.
• The operational definition should be applied consistently: if “all WHO interventions at least once” is the criterion, then the effective coverage proportion should reflect the lowest uptake intervention (e.g., 8.2%).

**Response:** this is the least proportion for all recommended services give at each visit and not considered as a measure of Effective coverage.
Recommendations:
1. Clarify the operational definition of effective coverage — is it “all WHO interventions at least once” or “some interventions at least once”?

**Response:** All intervention at least once. See table 4.
2. Ensure consistency between the definition and the reported results.

**Response:** we checked the entire document and consistent.
3. Revise the Results section to avoid contradictory figures (e.g., 25.9% vs. 8.2%).

**Response:** we have revised and tried to make self-explanatory. See table 4 of the revised manuscript.
4. Consider presenting both crude coverage (ANC 4+) and effective coverage (ANC 4+ plus all WHO interventions) separately, with clear explanation.

**Response:** thank you, corrected accordingly.
Discussion:
The discussion appropriately contextualizes findings within existing literature and offers plausible explanations for discrepancies.
To strengthen the impact of your manuscript, please revise the Discussion to move beyond restating the Results. Instead, focus on interpreting the clinical and public health significance of your key findings. For each major result, please address:
1. What does this mean for practice? (e.g., How should the very low rate of Gestational Diabetes Mellitus screening change clinical protocols or health worker training?)

**Response:** Thank you very interesting concern. See line 320-329 of the revised manuscript.
2. What are the policy implications? (e.g., Does the finding that women with prior complications receive better coverage suggest a need to reallocate resources or change messaging to reach low-risk women?)

**Response:** thank you, very interesting concern. See line 351-361.
3. How can these findings inform action? Conclude with targeted, practical recommendations for health system managers and policymakers in your setting."

Response: very interesting concern, see the revised manuscript conclusion.
Question: The finding that prior complications increase effective coverage suggests that risk-based care is occurring. Does this imply that low-risk women are being neglected? This has important programmatic implications.

**Response:** It doesn’t mean low risk mothers are being neglected rather those mothers who had learned from experience and well counseled get more effective coverage. Therefore, there is counseling limitation for pregnant women during their conception.

Generally, we have learnt a lot from your comments and we have tried to address all concerns raised by the reviewers.
