# Supplementary material for: Effective coverage and associated factors of antenatal care service among women who attended antenatal care at West Gojjam Zone Public Hospitals, Northwest Ethiopia
Source: PLoS One. 2026 Apr 21;21(4):e0347341. doi: 10.1371/journal.pone.0347341 (PMC13098960; doi:10.1371/journal.pone.0347341)
Supplement: Peer review history

# Peer review: Decision Letter 0

**DOI:** 10.1371/journal.pone.0347341.r001

**Authors:** Agegnehu Bante

13 Feb 2025

Dear Mr. Endalew,

Please submit your revised manuscript by Mar 30 2025 11:59PM. If you will need more time than this to complete your revisions, please reply to this message or contact the journal office at plosone@plos.org. When you're ready to submit your revision, log on to https://www.editorialmanager.com/pone/ and select the 'Submissions Needing Revision' folder to locate your manuscript file.. When you're ready to submit your revision, log on to https://www.editorialmanager.com/pone/ and select the 'Submissions Needing Revision' folder to locate your manuscript file.. When you're ready to submit your revision, log on to https://www.editorialmanager.com/pone/ and select the 'Submissions Needing Revision' folder to locate your manuscript file.. When you're ready to submit your revision, log on to https://www.editorialmanager.com/pone/ and select the 'Submissions Needing Revision' folder to locate your manuscript file.

- A rebuttal letter that responds to each point raised by the academic editor and reviewer(s). You should upload this letter as a separate file labeled 'Response to Reviewers'.
- A marked-up copy of your manuscript that highlights changes made to the original version. You should upload this as a separate file labeled 'Revised Manuscript with Track Changes'.
- An unmarked version of your revised paper without tracked changes. You should upload this as a separate file labeled 'Manuscript'.

If you would like to make changes to your financial disclosure, please include your updated statement in your cover letter. Guidelines for resubmitting your figure files are available below the reviewer comments at the end of this letter.

If applicable, we recommend that you deposit your laboratory protocols in protocols.io to enhance the reproducibility of your results. Protocols.io assigns your protocol its own identifier (DOI) so that it can be cited independently in the future. For instructions see: https://journals.plos.org/plosone/s/submission-guidelines#loc-laboratory-protocols. Additionally, PLOS ONE offers an option for publishing peer-reviewed Lab Protocol articles, which describe protocols hosted on protocols.io. Read more information on sharing protocols at . Additionally, PLOS ONE offers an option for publishing peer-reviewed Lab Protocol articles, which describe protocols hosted on protocols.io. Read more information on sharing protocols at . Additionally, PLOS ONE offers an option for publishing peer-reviewed Lab Protocol articles, which describe protocols hosted on protocols.io. Read more information on sharing protocols at . Additionally, PLOS ONE offers an option for publishing peer-reviewed Lab Protocol articles, which describe protocols hosted on protocols.io. Read more information on sharing protocols at https://plos.org/protocols?utm_medium=editorial-email&utm_source=authorletters&utm_campaign=protocols....

We look forward to receiving your revised manuscript.

Kind regards,

Agegnehu Bante

Academic Editor

PLOS ONE

Journal Requirements:

When submitting your revision, we need you to address these additional requirements.

1. Please ensure that your manuscript meets PLOS ONE's style requirements, including those for file naming. The PLOS ONE style templates can be found at https://journals.plos.org/plosone/s/file?id=wjVg/PLOSOne_formatting_sample_main_body.pdf and

https://journals.plos.org/plosone/s/file?id=ba62/PLOSOne_formatting_sample_title_authors_affiliations.pdf

2. We note that your Data Availability Statement is currently as follows: All relevant data are within the manuscript and its Supporting Information files.

Please confirm at this time whether or not your submission contains all raw data required to replicate the results of your study. Authors must share the “minimal data set” for their submission. PLOS defines the minimal data set to consist of the data required to replicate all study findings reported in the article, as well as related metadata and methods (https://journals.plos.org/plosone/s/data-availability#loc-minimal-data-set-definition).

For example, authors should submit the following data:

- The values behind the means, standard deviations and other measures reported;

- The values used to build graphs;

- The points extracted from images for analysis.

Authors do not need to submit their entire data set if only a portion of the data was used in the reported study.

If your submission does not contain these data, please either upload them as Supporting Information files or deposit them to a stable, public repository and provide us with the relevant URLs, DOIs, or accession numbers. For a list of recommended repositories, please see https://journals.plos.org/plosone/s/recommended-repositories.

If there are ethical or legal restrictions on sharing a de-identified data set, please explain them in detail (e.g., data contain potentially sensitive information, data are owned by a third-party organization, etc.) and who has imposed them (e.g., an ethics committee). Please also provide contact information for a data access committee, ethics committee, or other institutional body to which data requests may be sent. If data are owned by a third party, please indicate how others may request data access.

[Note: HTML markup is below. Please do not edit.]

Reviewers' comments:

Reviewer's Responses to Questions

**Comments to the Author**

1. Is the manuscript technically sound, and do the data support the conclusions?

Reviewer #1: No

Reviewer #2: Yes

**********

2. Has the statistical analysis been performed appropriately and rigorously?

Reviewer #1: No

Reviewer #2: Yes

**********

3. Have the authors made all data underlying the findings in their manuscript fully available?

The PLOS Data policy requires authors to make all data underlying the findings described in their manuscript fully available without restriction, with rare exception (please refer to the Data Availability Statement in the manuscript PDF file). The data should be provided as part of the manuscript or its supporting information, or deposited to a public repository. For example, in addition to summary statistics, the data points behind means, medians and variance measures should be available. If there are restrictions on publicly sharing data—e.g. participant privacy or use of data from a third party—those must be specified.requires authors to make all data underlying the findings described in their manuscript fully available without restriction, with rare exception (please refer to the Data Availability Statement in the manuscript PDF file). The data should be provided as part of the manuscript or its supporting information, or deposited to a public repository. For example, in addition to summary statistics, the data points behind means, medians and variance measures should be available. If there are restrictions on publicly sharing data—e.g. participant privacy or use of data from a third party—those must be specified.

Reviewer #1: Yes

Reviewer #2: Yes

**********

4. Is the manuscript presented in an intelligible fashion and written in standard English?

Reviewer #1: Yes

Reviewer #2: Yes

**********

Reviewer #1: 1. A revision of Abstract is mandatory, because of too much words and irrelevant result.

2. since revisionof result is needed, it is too difficult to conclude based on the above findings.

3. revision of background section is also seems mandatory because, most of the introduction section deems methods section

4. who were included and excluded in this study?

5. some operational difinitions need clarification

6. If the study was retrospective, would the authors revise the title and study design stated above???

7. How did yous assessed independent variables from a chart recorded, for instance distance from health facilities??

8. I advise to revise the entire document, especially result and discussion section again.

Reviewer #2: Line 83 "one third of one million" is a very unorthodox means of expressing a number. Please convert to the correct numbber

In table 2 it is not clear whether the complications refer to a previous or the current pregnancy. Pleaee make this clear.

To the reader when you discuss and present results concerning effective coverage it is confusing because for the various interventions, not all are recommended to be done at each visit. For example, ultrasound is recommended but nornmally only to be done once, whereas blood pressure ought to be meansured at each encounter. I think you need to include a clear description of the various essential interventions together with a description as to how many times it is recommended each are done throughout a pregnancy.

This could then be incorporated into Table 4 for greater clarity

The limitations section is incomplete and stops mid-sentence (lines 202-203) There are several more limitations that should be mentioned.

The discussion should be expanded to include some consideration as to the limitations of so called "effective care". Fully effective care includes not simply taking measurements but also acting upon them if the results give cause. For example, taking a blood pressure measurement is not effective unless there is evidence that high blood pressure receives appropriate attention and subsequent action to treat the problem.Whilst you might not have enough data available to measure this it is still a very important point to discuss and I would like you to do so.

**********

what does this mean?). If published, this will include your full peer review and any attached files.). If published, this will include your full peer review and any attached files.). If published, this will include your full peer review and any attached files.). If published, this will include your full peer review and any attached files.

If you choose “no”, your identity will remain anonymous but your review may still be made public.

**Do you want your identity to be public for this peer review?** For information about this choice, including consent withdrawal, please see our For information about this choice, including consent withdrawal, please see our For information about this choice, including consent withdrawal, please see our For information about this choice, including consent withdrawal, please see our Privacy Policy..

Reviewer #1: No

Reviewer #2: No

**********

[NOTE: If reviewer comments were submitted as an attachment file, they will be attached to this email and accessible via the submission site. Please log into your account, locate the manuscript record, and check for the action link "View Attachments". If this link does not appear, there are no attachment files.]

While revising your submission, please upload your figure files to the Preflight Analysis and Conversion Engine (PACE) digital diagnostic tool, https://pacev2.apexcovantage.com/. PACE helps ensure that figures meet PLOS requirements. To use PACE, you must first register as a user. Registration is free. Then, login and navigate to the UPLOAD tab, where you will find detailed instructions on how to use the tool. If you encounter any issues or have any questions when using PACE, please email PLOS at . PACE helps ensure that figures meet PLOS requirements. To use PACE, you must first register as a user. Registration is free. Then, login and navigate to the UPLOAD tab, where you will find detailed instructions on how to use the tool. If you encounter any issues or have any questions when using PACE, please email PLOS at . PACE helps ensure that figures meet PLOS requirements. To use PACE, you must first register as a user. Registration is free. Then, login and navigate to the UPLOAD tab, where you will find detailed instructions on how to use the tool. If you encounter any issues or have any questions when using PACE, please email PLOS at . PACE helps ensure that figures meet PLOS requirements. To use PACE, you must first register as a user. Registration is free. Then, login and navigate to the UPLOAD tab, where you will find detailed instructions on how to use the tool. If you encounter any issues or have any questions when using PACE, please email PLOS at figures@plos.org. Please note that Supporting Information files do not need this step.. Please note that Supporting Information files do not need this step.. Please note that Supporting Information files do not need this step.. Please note that Supporting Information files do not need this step.

# Author comment: Author response to Decision Letter 1

**DOI:** 10.1371/journal.pone.0347341.r002

7 Apr 2025

Detail point bay point response is submitted for reviewers

Attachment Submitted filename: Point by point response letter.docx

# Peer review: Decision Letter 1

**DOI:** 10.1371/journal.pone.0347341.r003

**Authors:** Agegnehu Bante

28 Jul 2025

Dear Dr. Endalew,

Thank you for submitting your manuscript to PLOS ONE. After careful consideration, we feel that it has merit but does not fully meet PLOS ONE’s publication criteria as it currently stands. Therefore, we invite you to submit a revised version of the manuscript that addresses the points raised during the review process.

Please submit your revised manuscript by Sep 11 2025 11:59PM. If you will need more time than this to complete your revisions, please reply to this message or contact the journal office at plosone@plos.org. When you're ready to submit your revision, log on to https://www.editorialmanager.com/pone/ and select the 'Submissions Needing Revision' folder to locate your manuscript file.. When you're ready to submit your revision, log on to https://www.editorialmanager.com/pone/ and select the 'Submissions Needing Revision' folder to locate your manuscript file.. When you're ready to submit your revision, log on to https://www.editorialmanager.com/pone/ and select the 'Submissions Needing Revision' folder to locate your manuscript file.. When you're ready to submit your revision, log on to https://www.editorialmanager.com/pone/ and select the 'Submissions Needing Revision' folder to locate your manuscript file.

- A rebuttal letter that responds to each point raised by the academic editor and reviewer(s). You should upload this letter as a separate file labeled 'Response to Reviewers'.
- A marked-up copy of your manuscript that highlights changes made to the original version. You should upload this as a separate file labeled 'Revised Manuscript with Track Changes'.
- An unmarked version of your revised paper without tracked changes. You should upload this as a separate file labeled 'Manuscript'.

If you would like to make changes to your financial disclosure, please include your updated statement in your cover letter. Guidelines for resubmitting your figure files are available below the reviewer comments at the end of this letter.

If applicable, we recommend that you deposit your laboratory protocols in protocols.io to enhance the reproducibility of your results. Protocols.io assigns your protocol its own identifier (DOI) so that it can be cited independently in the future. For instructions see: https://journals.plos.org/plosone/s/submission-guidelines#loc-laboratory-protocols. Additionally, PLOS ONE offers an option for publishing peer-reviewed Lab Protocol articles, which describe protocols hosted on protocols.io. Read more information on sharing protocols at . Additionally, PLOS ONE offers an option for publishing peer-reviewed Lab Protocol articles, which describe protocols hosted on protocols.io. Read more information on sharing protocols at . Additionally, PLOS ONE offers an option for publishing peer-reviewed Lab Protocol articles, which describe protocols hosted on protocols.io. Read more information on sharing protocols at . Additionally, PLOS ONE offers an option for publishing peer-reviewed Lab Protocol articles, which describe protocols hosted on protocols.io. Read more information on sharing protocols at https://plos.org/protocols?utm_medium=editorial-email&utm_source=authorletters&utm_campaign=protocols....

We look forward to receiving your revised manuscript.

Kind regards,

Agegnehu Bante

Academic Editor

PLOS ONE

Journal Requirements:

If the reviewer comments include a recommendation to cite specific previously published works, please review and evaluate these publications to determine whether they are relevant and should be cited. There is no requirement to cite these works unless the editor has indicated otherwise.

[Note: HTML markup is below. Please do not edit.]

Reviewers' comments:

Reviewer's Responses to Questions

**Comments to the Author**

Reviewer #3: (No Response)

Reviewer #4: (No Response)

Reviewer #5: (No Response)

Reviewer #6: (No Response)

**********

2. Is the manuscript technically sound, and do the data support the conclusions?

Reviewer #3: Partly

Reviewer #4: Yes

Reviewer #5: Partly

Reviewer #6: No

**********

3. Has the statistical analysis been performed appropriately and rigorously?

Reviewer #3: Yes

Reviewer #4: Yes

Reviewer #5: No

Reviewer #6: No

**********

4. Have the authors made all data underlying the findings in their manuscript fully available?

The PLOS Data policy requires authors to make all data underlying the findings described in their manuscript fully available without restriction, with rare exception (please refer to the Data Availability Statement in the manuscript PDF file). The data should be provided as part of the manuscript or its supporting information, or deposited to a public repository. For example, in addition to summary statistics, the data points behind means, medians and variance measures should be available. If there are restrictions on publicly sharing data—e.g. participant privacy or use of data from a third party—those must be specified.requires authors to make all data underlying the findings described in their manuscript fully available without restriction, with rare exception (please refer to the Data Availability Statement in the manuscript PDF file). The data should be provided as part of the manuscript or its supporting information, or deposited to a public repository. For example, in addition to summary statistics, the data points behind means, medians and variance measures should be available. If there are restrictions on publicly sharing data—e.g. participant privacy or use of data from a third party—those must be specified.

Reviewer #3: Yes

Reviewer #4: Yes

Reviewer #5: No

Reviewer #6: Yes

**********

5. Is the manuscript presented in an intelligible fashion and written in standard English?

Reviewer #3: No

Reviewer #4: No

Reviewer #5: Yes

Reviewer #6: No

**********

Reviewer #3: Manuscript title:

Effective Coverage and Associated Factors of Antenatal Care Service among Women Who Attended Antenatal Care at West Gojjam Zone Public Hospitals, Northwest Ethiopia

Competing interests: I have no competing interest.

This manuscript addresses a critically important topic: the effective coverage of antenatal care (ANC) services in a specific region of Ethiopia. The study's focus on "effective coverage," which integrates need, utilization, and quality, is a valuable approach. The findings highlight a concerningly low effective coverage rate and identify several associated factors. While the topic is highly relevant and the study design is appropriate for its stated aim, the manuscript has some methodological and reporting clarity issues that need to be addressed before publication.

The document exhibits several writing issues that should be addressed. A thorough proofread by a native English speaker or a professional editing service would be highly beneficial. Focus specifically on:

• Correcting all "data was" to "data were."

• Ensuring all percentages in the "Results" section have clearly defined and correct denominators.

• Reviewing interpretations in the "Discussion" for logical consistency with the findings.

• Improving transitions between sentences and paragraphs.

• Adding the crucial missing limitations (selection bias from population definition, short study period).

• Refining precision in word choice and sentence structure throughout.

Please find the attached file for my point by point comments .

Reviewer #4: Abstract: There are a few points that would help sharpen the focus of the abstract. For example, in the phrasing “due to ineffective coverage of antenatal care service and other reason” (line 17), “other reasons” should be specified (e.g., poor health infrastructure, socioeconomic barriers) or removed to sharpen the focus. The phase (line 19) “Despite, there is limited information on effective coverage of Antenatal care in Ethiopia” is grammatically incorrect and should be revised (such as, “However, there is limited evidence on the effective coverage of antenatal care in Ethiopia”). The abstract would also benefit from an overall grammatical and stylistic editing, such as inconsistent capitalization (antenatal and Antenatal – line 18 versus line 20) t improve the overall clarity.

Introduction: The introduction addresses the critical health issue of maternal mortality and introduces “effective coverage” as a key health system performance metric that integrates the concepts of need, utilization, and quality. This is a highly relevant concept, particularly in the context of maternal and antenatal care (ANC) in low-resource settings. It is also important to see continued work in better measurement of quality-related concepts versus numbers-only indicators to truly measure progress.

In general, it may help to strengthen the introduction to reverse the current order of ideas so that the argument cascades logically from the global to specific. The narrative currently starts at the micro-level by explaining ANC’s benefits, and then shifts abruptly to global inequities with the EC framework concepts interspersed. The ideas are already there, but a more logical sequence of the global-to-specific would provide a more logical sequence would build a stronger case for the reader. It will also help readers more readily understand the study’s rationale and the added value of examining effective coverage specifically in the Ethiopian context.

For example, you could consider opening with the striking global statistics on maternal mortality, highlighting women in low- and middle-income settings face a higher burden than those in high-income settings (lines 81-85). Then moving to the more specific situation in the Ethiopian setting which provides the context for this study (lines 85-90). This can help establish up front the scale and urgency of the problem. From there, narrowing to the focus on ANC as one of the most important evidence-based interventions for detecting and averting life threatening maternal conditions. This shows readers why ANC quality matters in solving this global challenge (lines 48-57; lines 91-97). Once this context has been established, then you could introduce the concept of effective coverage as the lens through which the study investigates not merely if women attended ANC and enough sessions of ANC, but whether or not they received the quality of services needed (starting with line 58 and remaining sections not cited above).

There are also some points where some adjustments to phrasing would be helpful to more clearly convey the concepts. For example, introducing the concept of effective coverage (line 54), while it is conceptually strong, would benefit from clearer phrasing, such as “Effective coverage is a composite indicator of health system performance…” or perhaps other phrasing that would more clearly express this concept and the intended meaning. Related, there are some circular sentence structures. For example, “use refers to the use of services” (line 58) which offers no additional meaning as is.

There are also some confusing or differing definitions. For example, quality is first defined as “the actual health benefit experienced from the service” (lines 58-59), but later operationalized in terms of receiving the recommended content (lines 62-64). Clarifying this conceptual leap would help improve the flow and strengthen the case you are building.

The formula (lines 69-70) is a bit hard to read that just appears to be a formatting issue. Ensuring the equation is formatted more clearly would be helpful to improve comprehension.

Another point where the phrasing could be improve is the study objective (lines 101-103). While the objective is clearly stated and aligns well with the background, the wording could be more concise and grammatically polished, such as “The study aimed to assess he effective coverage of antenatal care and identify associated factors among women attending ANC at public hospitals in West Gojjam Zone, North west Ethiopia.”

It would also strengthen your argument to provide some more evidence of the situation in Ethiopia around lines 87-90 – such as regional disparities, policy interest in quality improvement, and existing studies (or lack thereof the highlight an important knowledge gap that your study helps to address).It would also help strengthen how this study addresses a knowledge gap by providing some examples of prior studies that focused on crude coverage or perception rather than EC and that none examined the specific zone of the study in order to justify the study’s objective more convincingly.

There are some areas where citations are needed, such as the maternal death statistics provided in lines 67-70. There are also some grammatical and stylistic errors that detract from the content. Line 73-76 has a run-on sentence that could be adjusted to two sentences: One possible reason for the persistently high maternal mortality rate could be the poor quality of care, which is not reflected in crude coverage measures. Additionally, the poor uptake of essential interventions during routine ANC visits may have contributed to the stagnant or slow rate of reduction in maternal mortality.

In summary, the content is relevant and valuable but needs improved logical flow, clearer definitions, accurate equation formatting, and some careful language editing to convey the message more effectively.

Methods: The methods section provides a clear overview and includes all the required elements of the study design, setting, and sampling approach. However, several issues reduce the clarity and rigor that can be addressed. Tightening the language, clarifying the sampling procedure, and improving the structure for readability and methodological transparency would benefit this section. Also, please check the size and font of the headings. It seems that everything up through sample size is other “Methods” heading and the remaining (data management, data quality, etc.) under the “Measurement and Variables” heading. It is a bit confusing if this was what was intended.

Study design and setting – The setting is well described but is overall detailed regarding geographic distances and hospital names. This could be condensed for focus and readability.

Population – The phrases “the study population of this study” are redundant (lines 124 and 126). It is also unclear whether “women who completed ANC follow-up” (line 127) means they had all four recommended visits (line 125) that is cited before or a different criterion.

Sample size determination and sampling procedures – The sample size calculation is methodologically sound but presented with inconsistent formatting and minor typographical errors (such as line 122 “1.5 design effect” lacks spacing). Consider rephrasing the formula and description of what was used to enhance clarity – perhaps start with the formula that was used, justifying a single population proportion formula, and then the assumptions used for the calculation.

The multi-stage random sampling approach is appropriate but the description could be clarified. For example, the phrase “multi-stage simple random sampling” (line 138) is confusing as multi-stage sample and simple random sampling are typically distinct. A more precise explanation of each stage would help. It would also be helpful to note how confidentiality and randomness were ensured with the use of the medical registration numbers (lines 140-142).

Measurement and variables – While this section presents the outcome and explanatory variables clearly in general terms, it would also benefit from further clarity, organization, and precision.

The initial sentence outlining the outcome variable (effective coverage of ANC) is appropriate, but the paper would benefit from further clarity in the definition. The text describes this further in lines 152-156 and notes that women with four or more ANC visits who received “WHO recommended interventions at least once” were considered to have achieved effective coverage. However, this definition is vague in its treatment of timing and frequency. It is unclear whether receiving the interventions only once is considered sufficient, even when guidelines may emphasize repeat or time-specific administration. The list of WHO-recommended interventions (lines 157-160) is presented in a run-on sentence and should be formatted as a clear, concise list to aid readability.

But the subsequent listing of independent variables is dense, inconsistently phrased, and lacks logical structure (lines 144-151). The grouping is also confusing, with some sociodemographic variables and some infrastructure variables. Clinical and facility-level variables are intermingled without sub-categorization, which makes this section hard to follow. Organizing by thematic groups (individual-level, clinical history, health system/infrastructure) would significantly improve readability and comprehension.

There are a number of abbreviations that are not defined - PROM, APH, PIH, PPH (line 147); PID (line 148); SFH (line 157); HCT,VDRL, BG^RH,U/A (lines 161-162); IESO (line 164); and BP (line 166).

The language used throughout this section is often vague or awkward. Terms such as “Gynecology related factors” (line 146) and “Investigation and infrastructure related variables” (lines 148-149) lack specificity and are not clearly defined. Additionally, the phrase “presence of adequate trained human power” (line 150) is grammatically incorrect and could be replaced with more natural phrasing such as “availability of adequately trained personnel.”

There are also typographical errors that detract from the professionalism of the text, such as “pervious History of Gynecology Complication,” (line 148) which should read “previous history of gynecological complications.” Such errors can undermine the credibility of the methodological description.

Data collection tools and procedure - This section provides basic information on the data collection tools and procedures but lacks sufficient detail and clarity. While it notes that data were collected using an adapted structured questionnaire translated from English to Amharic, it does not specify the source of the questionnaire, the nature of the adaptation, or whether the tool was pretested or validated. Additionally, the description of data collectors and supervisors is brief and imprecise; stating that three diploma and two BSc nurses were recruited is helpful, but it would be more informative to mention any training they received, how data quality was ensured, and how supervision was conducted.

Data quality control - The description in this section is quite general and could benefit from greater specificity. For example, it is unclear what specific adjustments were made following the pretest, or how issues of reliability and data entry accuracy were handled. Additionally, a one-day training duration seems insufficient for complex data collection tasks, especially if the questionnaire included technical or clinical components. More detail on the rationale for that limited training or the quality assurance during data collection and entry to minimize bias or errors would strengthen the credibility of this section.

Data management and analysis - This section needs clearer language and technical precision. There are grammatical errors (e.g., “cheeked” should be “checked” on line 185) and unclear phrasing around model fit. Standard terms like bivariate versus bivariable; multivariate versus multivariable should be used consistently. While the use of p-values to select variables is appropriate, the rationale and handling of issues like multicollinearity or missing data are not discussed. Overall, the section covers key steps but requires clearer writing and more detail for full transparency.

Limitation of this study – There is an incomplete sentence here. This section should be reviewed and ensure the full limitations are discussed. Other factors such as the cross-sectional design, possible issues with record-keeping, lack of timing for interventions may also be important limitations to discuss in this section.

Overall, while this section contains the essential elements required for transparency, it would benefit greatly from improved structure, clearer language, and more thoughtful integration of supporting references. Some sections are underdeveloped and require further elaboration to demonstrate the reliability and rigor of the data collection and analysis processes.

Results: The data presented are relevant, the section would benefit from clearer writing and a more logical flow. Examples of some phrasing and formatting that needs improvement, includes:

• “We analyzed and included in the final analysis…” (line 206) could be simplified for clarity, such as “We analyzed 680 ANC service records”;

• “almost two third” (line 208) should be “almost two-thirds”; also found in line 214

• line 212-214 could be rephrased, “More than half the women were multipara (56.8%), followed by grand multipara (17.8%)”

• the “distance from HF” row is missing a parenthesis after 14.1

• spacing between numbers and parenthesis should be consistent (e.g., Table 2 – some are presented with a space 85 (12.5) and some without 386(56.8)). This is found in the remaining tables.

• Data presentation should be consistent – most present 1 digit, but line 216 presents 2 digits (48.18%)

• Line 217 should read “obstetric complications” or perhaps better rewritten “Among women who experienced obstetric complications during pregnancy, nearly half (48.2%) had pregnancy-induced hypertension, followed by antepartum hemorrhage (32.1%)”

• Line 228-229 – select consistent capitalization for Hospital or hospital.

Similar suggestions are found throughout the remaining results section and should continue to check for this consistency in presentation, logical flow, and clarity of presentation.

Descriptions of the results presented in the tables could be improved. For example, in the description for Table 1, the text notes that 72.9% were in the age range 21-35 but the table shows groups 15-24, 25-34, and 35 or more. The description should match the way data is presented. If there is a reason for discussing results differently than how it is presented in the table, this should be explained.

The description for Table 4 of very unclear, as is why the CI is presented in text and not the table. Are these univariates among all 680 women who had four or more visits? It is also unclear where the description of “80% of mothers were diagnosed using ultrasound” is coming from (line 237-238).

The section around table 5, which presents findings from bi-variable and multivariable logistic regression, lacks clarity, consistency, and precision in language, which makes it a bit hard to follow. The opening sentence is overly long and awkwardly constructed. Phrases like “identified as a candidate variables” (line 246) contain both grammatical and pluralization errors. It would benefit from clearer subject-verb agreement and tighter phrasing. The use of "multiple logistic regression" should be replaced with the standard term "multivariable logistic regression" for accuracy.

Additionally, the narrative interpretation of odds ratios is not always intuitive or clearly explained. For example, stating that the "odds were 68% lower" when the AOR is 0.32 may confuse readers unfamiliar with interpreting odds ratios. This could be better framed as "women aged 25–34 had significantly lower odds of effective coverage compared to those aged 15–24."

There is also some repetition and awkward phrasing. For example, "those mothers who had no encountered previous gynecological complications" (lines 260-261) should be revised for grammatical accuracy ("those who had not experienced..."). Finally, references to “table 5” should ideally be integrated more naturally into the narrative or visually indicated in the formatting, especially when listing statistical results.

Overall, the results section communicates important findings but would benefit from clearer language, grammatical revision, and improved statistical interpretation.

Discussion: The discussion summarizes key findings but is weakened by problems in structure, logic, and writing quality. There is a lot of repeating of the results but no interpretation of those results is provided.

The opening paragraph contains an incomplete phrase - “The overall effective coverage of ANC services 25.9%” (line 270) and immediately compares results with previous studies without first interpreting their substantive meaning of the result found in this study. Comparisons to earlier Ethiopian and Pakistani studies are offered, yet the explanations (“differences in social backgrounds” or “where the study was done”) are speculative and unsupported by data. Citing concrete contextual factors from these comparative studies (e.g., sampling frame, facility type, survey year) would be more convincing and better ground your argument.

Throughout, percentages are repeated without context, interpretation, and discussion. For example, readers are told that SFH measurement was 100% (line 278) and that gestational diabetes screening was 8.2% (line 281). But what this means in the Ethiopian – or larger – context or the implications for maternal outcomes or service quality are not discussed. This fails to help the reader understand the significance of these findings.

Interpretations of odds ratios are sometimes inaccurate or poorly framed. For example, describing the adjusted odds ratio of 0.32 for women aged 25–34 as “68% lower” is mathematically correct (line 290), yet no rationale is provided for why younger women achieve higher effective coverage. Speculation about “active reproductive group level” (line 292) lacks clarity and evidence.

Similarly, attributing higher coverage among multiparous women to “more exercise about its follow up” (line 297) is vague and grammatically incorrect. The discussion routinely labels findings as “contralateral” or “contrast,” but it fails to explore potential explanations such as health seeking behavior, facility readiness, or cultural norms. Other grammatically confusing or incorrect sentences can be found throughout as well - (“this could be that this age group is active reproductive group level” (lines 291-292); “women who had multiparty have more exercise about its follow up” (line 293-294).

Overall, a more effective approach for the discussion section would be to highlight the most salient associations, relate them to existing literature, and consider programmatic implications. This section also requires clearer writing, stronger linkage between results and interpretation, and evidence based explanations for disparities.

Conclusion: The conclusion lacks a strong impact. It requires refinement to offer a more coherent and policy-relevant summary of the study’s implications.

Reviewer #5: Thank you for the opportunity to review your manuscript. This is an important topic and could have a major impact on maternal and perinatal mortality. The study estimated the effective coverage of antenatal care in women who had at least 4 AN appointments. The medical records of 680 women who attended antenatal care from 30 June 2023 to 30 July 2023 in one of 3 randomly selected hospitals out of the 8 in West Gojjam Northwest Ethiopia were reviewed by trained data collectors.

The study aims to address an important issue and provides valuable estimates of the rates of crude and effective coverage. However there are some significant limitations with the statistical analysis.

Abstract - I struggled to match the CIs in lines 37-40 with adjusted odds ratios presented in table 5.

Main text - Consideration of Selection bias - How well did the study population match the source population in terms of demographics? Did the criteria of requiring 4 ANC affect recruitment? One of the hospitals selected was the larger district hospital - are the women who attend this larger hospital comparable to those attending the 2 smaller local hospitals?

The tables and figure titles should be clearer eg Table 1: shows the demographics of pregnant women in the 3 hospitals in the study, not the region. Rather than referring to the region, these tables and figures should referring to 640 women with ante-natal care in 3 hospitals in the province, during one month June- July 2023

Effective coverage only shown in total and not by hospital, but there were clear differences in crude coverage across the 3 hospitals. I would recommend that hospital should be included in the regression modelling in separate models, as a fixed effect but also in a multilevel model as a random effect, to explore within and between hospital variance. This impact of this and confounding across variables mean the results of the multivariable regression presented are likely unstable. Was the likely confounding across many of the variables assessed? Of particular concern are estimates that have changed direction between the bivariate and multivariate analyses; not all of these variables are appropriate to be included in the same model. eg age is confounded with multiple variables including parity, planned pregnancy and marital status.

The discussion would benefit from expansion and should include reference to selection bias and limitations in the statistical analyses.

Reviewer #6: The authors present an interesting research question aimed at quantifying effective antenatal care and they propose to examine multiple variables that may impact care. Unfortunately, the manuscript lacks any sound conclusions, presents inconsistent data, and has numerous typographical and grammar errors making it unsuitable for publication at this time. The most concerning problem with this manuscript is that the data the authors present is inconsistent throughout, leaving the reader unclear what is truth and how that impacts their research question. For example, in the manuscript’s abstract the authors cite “Parity (multipara) (AOR: 1.74; 95 % CI:0.05- 0.55)” but that data is not repeated or presented anywhere in the paper that follows. The main source of this data is in Table 5, but the numbers in that table are different from what’s stated in the abstract. This happens for multiple of the key variables they explored.

Also, many of their AORs and CIs don’t make sense where the AOR does not fall within the 95% CI. This is seen in the “Parity (multipara)” example stated above, the “previous gynecological complication (AOR:3.98; 95% CI; 1.33-3.70)” cited in the abstract, and also in the manuscript’s Results and Discussion sections.

Finally, the conclusions reached in this manuscript aren’t actually conclusions, but statements about the data. It’s not meaningful to just state which variables are significantly associated with (in)effective coverage of ANC services, the authors should also explain if these variables positively or negatively impacted coverage. For example, did being married or divorced increase or decrease effective coverage? Both have positive AORs and highly variable CIs.

It is this reviewers recommendation to reject the manuscript in its current form. The authors need to revisit their data analysis and conclusions. Ideally, they would also have more editing done to remove the grammar and typographical mistakes make the writing easier to follow.

**********

what does this mean?). If published, this will include your full peer review and any attached files.). If published, this will include your full peer review and any attached files.). If published, this will include your full peer review and any attached files.). If published, this will include your full peer review and any attached files.

If you choose “no”, your identity will remain anonymous but your review may still be made public.

**Do you want your identity to be public for this peer review?** For information about this choice, including consent withdrawal, please see our For information about this choice, including consent withdrawal, please see our For information about this choice, including consent withdrawal, please see our For information about this choice, including consent withdrawal, please see our Privacy Policy..

Reviewer #3: No

Reviewer #4: No

Reviewer #5: No

Reviewer #6: **Yes:** Samantha ByrnesSamantha ByrnesSamantha ByrnesSamantha Byrnes

**********

[NOTE: If reviewer comments were submitted as an attachment file, they will be attached to this email and accessible via the submission site. Please log into your account, locate the manuscript record, and check for the action link "View Attachments". If this link does not appear, there are no attachment files.]

While revising your submission, please upload your figure files to the Preflight Analysis and Conversion Engine (PACE) digital diagnostic tool, https://pacev2.apexcovantage.com/. PACE helps ensure that figures meet PLOS requirements. To use PACE, you must first register as a user. Registration is free. Then, login and navigate to the UPLOAD tab, where you will find detailed instructions on how to use the tool. If you encounter any issues or have any questions when using PACE, please email PLOS at . PACE helps ensure that figures meet PLOS requirements. To use PACE, you must first register as a user. Registration is free. Then, login and navigate to the UPLOAD tab, where you will find detailed instructions on how to use the tool. If you encounter any issues or have any questions when using PACE, please email PLOS at . PACE helps ensure that figures meet PLOS requirements. To use PACE, you must first register as a user. Registration is free. Then, login and navigate to the UPLOAD tab, where you will find detailed instructions on how to use the tool. If you encounter any issues or have any questions when using PACE, please email PLOS at . PACE helps ensure that figures meet PLOS requirements. To use PACE, you must first register as a user. Registration is free. Then, login and navigate to the UPLOAD tab, where you will find detailed instructions on how to use the tool. If you encounter any issues or have any questions when using PACE, please email PLOS at figures@plos.org. Please note that Supporting Information files do not need this step.. Please note that Supporting Information files do not need this step.. Please note that Supporting Information files do not need this step.. Please note that Supporting Information files do not need this step.

Attachment Submitted filename: PONE_Review.docx

# Author comment: Author response to Decision Letter 2

**DOI:** 10.1371/journal.pone.0347341.r004

2 Aug 2025

Point by point response letter

Title: Effective Coverage and Associated Factors of Antenatal Care Service among Women Who Attended Antenatal Care at West Gojjam Zone Public Hospitals, Northwest Ethiopia

Reviewer #3: Competing interests: I have no competing interest.

This manuscript addresses a critically important topic: the effective coverage of antenatal care (ANC) services in a specific region of Ethiopia. The study's focus on "effective coverage," which integrates need, utilization, and quality, is a valuable approach. The findings highlight a concerningly low effective coverage rate and identify several associated factors. While the topic is highly relevant and the study design is appropriate for its stated aim, the manuscript has some methodological and reporting clarity issues that need to be addressed before publication.

The document exhibits several writing issues that should be addressed. A thorough proofread by a native English speaker or a professional editing service would be highly beneficial. Focus specifically on:

Correcting all "data was" to "data were."

Response: Thank you. It is corrected in the entire document.

Ensuring all percentages in the "Results" section have clearly defined and correct denominators.

Response: We wonder about this insight and corrected it in the revised manuscript.

Reviewing interpretations in the "Discussion" for logical consistency with the findings.

Response: Thank you. We found this comment very interesting and corrected accordingly.

Improving transitions between sentences and paragraphs.

Response: corrected accordingly.

Adding the crucial missing limitations (selection bias from population definition, short study period).

Response: We want to forward our great appreciation for the reviewer regarding these issues. The way we write the study population and study period was quite different from the actual study work while manuscript preparation. See the revised manuscript.

Refining precision in word choice and sentence structure throughout.

Response: Corrected accordingly.

Point by point comments

Abstract:

Reconsider the wording "Despite, there is limited information." It's a bit awkward. "Despite this, limited information exists..." or "However, information is limited..." would be smoother.

Response: thank you. Corrected accordingly. See the revised manuscript

Period: "from 30 June 2023 to 30 July 2023" is a very short period (one month). While the total sample size (680) is good, it's important to confirm this timeframe. If the study was conducted over such a brief period, consider if this has any implications for the representativeness of your sample.

Response: Thank You. The above period was the data extraction period while the exact study period was from September 01, 2021, to August 30, 2022. See the revised manuscript

Clarity of Factors: For categorical variables like "Age 15 to 35," "being married," "divorced," and "multipara," please clarify what the reference category was for the AORs. This is essential for interpreting the reported associations. For example, was "Age 15 to 35" compared to "Age <15" or "Age >35"? Was "married" compared to "single"?

Response: See table 6 of the revised manuscript. We state there.

Introduction:

Line 67-70: "It is estimated that every day in 2020, almost 800 women died, maternal death occurred almost every two minute and preventable causes related to pregnancy and childbirth result in the deaths of approximately 330,000 women worldwide every year."

Please check the consistency of these numbers. "Almost 800 women died every day" (800 x 365 days = 292,000 annually) and "almost every two minute" (which is 30 deaths per hour or 720 per day, equating to ~262,800 annually) seem to contradict the "approximately 330,000 women worldwide every year" statistic. While all are high, it's best to present a single, consistent figure or range with a clear citation for the most recent estimates (e.g., from WHO/UNFPA/World Bank reports) to avoid confusion.

Response: Thank you, important comment. See line 67 to 72 of the revised manuscript.

Line 71-72: "The maternal mortality rate in Ethiopia is high which is, 412 deaths per 100,000 live births” I don’t think this is a recent figure; try to refer recent report.

Response: very interesting concern. See line 72 to 76 of the revised manuscript.

Study design and settings:

Line 94: "An institutional-based analytical cross-sectional study design using secondary data was carried out..."

Comment: "Institutional based" is slightly redundant given you immediately specify "in public hospitals." Consider simplifying "A facility-based analytical cross-sectional study..."

Suggestion: "A facility-based analytical cross-sectional study was conducted, utilizing secondary data from public hospitals in West Gojjam Zone."

Response: Corrected accordingly.

Lines 95-96: "...from 30 June 2023 to 30 July 2023."

Major Comment: A one-month study period for a cross-sectional study, especially one using secondary data, is remarkably short. This raises significant concerns about the representativeness of the sample and the potential for seasonal variations in ANC attendance or "effective coverage" to be missed.

Response: The study period was from September 01, 2021, to August 30, 2022. See the revised manuscript study period.

Question for Authors: Why was such a short duration chosen? Was this sufficient to achieve the required sample size, considering the average monthly ANC attendance at these hospitals? How do you account for potential monthly or seasonal fluctuations in service utilization?

Response: The study period was from September 01, 2021, to August 30, 2022. See the revised manuscript study period

Lines 96-99: "West Gojjam is one of an administrative zone... As of 2019, the zone had a population of 2,106,596..."

Suggestion: Ensure consistency in the reference year for population data. If the study was 2023, why is the population data from 2019? Is there more recent data available, or is 2019 the latest available projection? Acknowledge this if it's the latest available.

Response: Thank you! corrected accordingly. See the revised manuscript.

Lines 103-107: "The average number of ANC4 visits per year at each hospital was as follows: Finote Selam General Hospital (1,197)... Dembecha Primary Hospital (2)."

Major Comment: The figure for Dembecha Primary Hospital (2 ANC4 visits per year) is an extreme outlier and highly problematic. This number is incredibly low for a primary hospital providing ANC services.

Response: Thank you very much! it was a typing error. There were 742 women who attended 4th ANC visit at Dembecha Hospital.

Population:

There are gross grammatical errors in defining your population please try to revise it.

Redundancy: your study population and inclusion criteria are a clear repetition.

Potential for Selection Bias: By focusing solely on women who completed at least four ANC visits within the study hospitals and excluding those who didn’t, your study population might be healthier, more compliant, or face fewer barriers to care than the general population of pregnant women. Please acknowledge this potential selection bias as a limitation

Response: the study population of study was all pregnant women conducting ANC follow-up at the Maternal and Child Health (MCH) units of selected West Gojjam zone public hospitals during the data collection period. But the former study population we wrote was taken from operational definition of Effective coverage due to a typing error while revising the author’s comment. See the revised manuscript

Sample size determination and sampling procedures

The formula should explicitly show how the design effect and non-response rate are incorporated.

The calculated sample size is approximately 688, not 680 based on your assumption, please justify

Response: thank you for your concern. But still the sample size calculation was correct for us. n= (〖Za/2)〗^2 (P)(1-P)/〖(d)〗^2 =1.96*1.96(0.22*.78)/(0.004)2

=3.84*0.22*0.78/0.0016=411.84 *1.5 design effect=617.76 +10% non-response rate (10*618) = 618+62=680 total sample size

Line 126: "The study participants were chosen using a multi-stage simple random sampling technique." This phrasing is slightly contradictory. It's a "multi-stage sampling technique," and then you specify the type of random sampling used at each stage.

Response: we use more than one stage and lastly, we apply simple random sampling to select the study participants medical registration /chart, as per our experience and level of knowledge such king of sampling technique= multi-stage simple random sampling technique.

How did you ensure that there were enough eligible women (those completing ANC4) within that specific one-month period in the selected hospitals to draw a sample of 688?

Response: same as above response. The study period was too long not one month.

What if the ANC4 completers in the 3 selected hospitals during June 30 - July 30, 2023, was less than 688

Response: same as above response. The study period was too long, not one month.

Did you checked the adequacy of the calculated sample size for your second objective? Factors associated with EC? You need to show that.

Response: thank you. It was checked, see table one of the revised manuscript.

Measurements and Variables

What does "Status of pregnancy" and “Abortion” refer? Clarify

Response: Thank you. See the revised manuscript, it is elaborated.

"Investigation and infrastructure related variables (presence of ultrasound, presence of baseline investigation for pregnant women, presence of adequate trained human power, presence of adequate equipment and supplies at ANC unit)" these are facility-level variables, not typically found in individual client charts.

How these variables were assessed, how were they operationalized and collected? this needs to be explained in the "Data Collection Tools and Procedures" section.

Response: Thank you very much! We were used as utilization variable by everyone except for the presence of adequate equipment and supplies at ANC unit variables. presence of adequate equipment and supplies at ANC unit variables were not used during the analysis of data.

Data collection tools and procedures

From where did you adapt the questionnaire? Was it based on a WHO checklist template or another established survey tool? Briefly mention.

Response: thank you. See the revised manuscript, we tried to clarify it.

Why do you need to translate the questionnaire? Were the medical records of the clients written in Amharic or the data collectors didn’t understand English?

Response: the questionnaire was prepared in English only. See the revised manuscript.

This part is very brief and lacks critical details needed for a PLOS ONE methods section.

Response: Thank you! see the revised manuscript.

Data management and analysis

How do you calculate EC? Briefly explain the analysis.

Response: As we elaborate in the operational definition section, we count mothers who had both “ANC4+ ANC visits and getting WHO recommended services”/ total sample size *100

Strict Separation of Methods and Results: remove the Hosmer-Lemeshow P-value and interpretation from the methods section.

Response: We corrected accordingly.

Grammar and Phrasing: Pay attention to subject-verb agreement ("data was" vs. "data were") and awkward phrasing to enhance professionalism and readability.

Response: Really appreciated comment and we correct through out the manuscript document. We also check the grammar of the whole manuscript document.

Detail for Data Cleaning: While not a major flaw, PLOS ONE encourages authors to briefly mention specific steps taken during data cleaning (e.g., how missing data were handled, checks for outliers). This adds to transparency.

Response: thank you. There was no missing data.

Ethical considerations

Was individual-informed consent obtained from each patient whose data was extracted? If not, was the requirement for informed consent waived by the ethics committee? If waived, what was the justification for this waiver (e.g., de-identified data, minimal risk, retrospective nature, impracticability of obtaining consent)?

Response: Thank you for your concern. We tried to elaborate more in the revised manuscript. Consent was waived due to its retrospective nature.

Results

Lines 202-203: "Almost half of women 63(48.18%) had pregnancy induced hypertension followed by APH 42(32.1%) from obstetric complication in pregnancy (Table 2)."

63 out of your total sample of 680 is 9.26%, not 48.18%.

Similarly, 42 out of 680 is 6.18%, not 32.1%.

Response: Thank you. We corrected it accordingly.

Lines 213-216: "Among women who attended their ANC services in Finoteselam General Hospital (74.7%), Feresbet Primary hospital (51.7%), and Liben primary hospital (67.9%), had received at least four ANC visits, even when timing and scheduling were not taken into account (Figure 1)."

Major Issue: Contradiction with Study Population. This sentence directly contradicts your "Population" section, which stated that your study population was defined as pregnant women who attended and completed at least four ANC visits.

Response: Thank you. This comment helps us to correct the methodological problems that we made during manuscript preparation in general. We want to acknowledge you again for your invaluable comments.

Discussion

Lines 274-278: "The odds of effective coverage of ANC among mothers with age group of 25-34 years were 68% lower than that of mothers with age group of 15-24years... This could be that this age group is active reproductive group level and had more awareness about the importance of ANC follow-up."

Major Logical Inconsistency: If the 25-34 age group is "more aware" and "active," logically one would expect higher (not lower) odds of effective coverage. Your explanation contradicts your finding.

Response: Thank you, we corrected it accordingly.

Limitations

Line 297: "Certain variables crucial for a comprehensive assessment…

Consider giving one or two specific examples of such variables

Acknowledge other limitations of your study such as:

Selection Bias due to Study Population Definition

Short Data Collection Period

Facility-Level Variables collected from Client Charts?

Response: Thank you! All the raised issues are interesting and addressed by this study.

Your article needs thorough revision and lots of editions. Please go through the whole document and edit grammar and language, spelling, and punctuation errors. Addressing these points will significantly improve the manuscript's overall quality and readability for publication.

Response: thank you very much for your interesting comments. We found your whole comments very important to improve our manuscript and we were happy to correct each comments raised. We believe this manuscript is well improved accordingly.

Reviewer #4: Abstract: There are a few points that would help sharpen the focus of the abstract. For example, in the phrasing “due to ineffective coverage of antenatal care service and other reason” (line 17), “other reasons” should be specified (e.g., poor health infrastructure, socioeconomic barriers) or removed to sharpen the focus.

Response: We appreciated this comment and corrected.

The phase (line 19) “Despite, there is limited information on effective coverage of Antenatal care in Ethiopia” is grammatically incorrect and should be revised (such as, “However, there is limited evidence on the effective coverage of antenatal care in Ethiopia”).

Response: Corrected in the revised manuscript.

The abstract would also benefit from an overall grammatical and stylistic editing, such as inconsistent capitalization (antenatal and Antenatal – line 18 versus line 20) t improve the overall clarity.

Response: Corrected accordingly in the revised manuscript.

Introduction: The introduction addresses the critical health issue of maternal mortality and introduces “effective coverage” as a key health system performance metric that integrates the concepts of need, utilization, and quality. This is a highly relevant concept, particularly in the context of maternal and antenatal care (ANC) in low-resource settings. It

Attachment Submitted filename: Point by point response 2.docx

# Peer review: Decision Letter 2

**DOI:** 10.1371/journal.pone.0347341.r005

**Authors:** Agegnehu Bante

28 Oct 2025

Dear Mr. Endalew,

Thank you for submitting your manuscript to PLOS ONE. After careful consideration, we feel that it has merit but does not fully meet PLOS ONE’s publication criteria as it currently stands. Therefore, we invite you to submit a revised version of the manuscript that addresses the points raised during the review process.

Please submit your revised manuscript by Dec 12 2025 11:59PM. If you will need more time than this to complete your revisions, please reply to this message or contact the journal office at plosone@plos.org. When you're ready to submit your revision, log on to https://www.editorialmanager.com/pone/ and select the 'Submissions Needing Revision' folder to locate your manuscript file.. When you're ready to submit your revision, log on to https://www.editorialmanager.com/pone/ and select the 'Submissions Needing Revision' folder to locate your manuscript file.. When you're ready to submit your revision, log on to https://www.editorialmanager.com/pone/ and select the 'Submissions Needing Revision' folder to locate your manuscript file.. When you're ready to submit your revision, log on to https://www.editorialmanager.com/pone/ and select the 'Submissions Needing Revision' folder to locate your manuscript file.

- A rebuttal letter that responds to each point raised by the academic editor and reviewer(s). You should upload this letter as a separate file labeled 'Response to Reviewers'.
- A marked-up copy of your manuscript that highlights changes made to the original version. You should upload this as a separate file labeled 'Revised Manuscript with Track Changes'.
- An unmarked version of your revised paper without tracked changes. You should upload this as a separate file labeled 'Manuscript'.

If applicable, we recommend that you deposit your laboratory protocols in protocols.io to enhance the reproducibility of your results. Protocols.io assigns your protocol its own identifier (DOI) so that it can be cited independently in the future. For instructions see: https://journals.plos.org/plosone/s/submission-guidelines#loc-laboratory-protocols. Additionally, PLOS ONE offers an option for publishing peer-reviewed Lab Protocol articles, which describe protocols hosted on protocols.io. Read more information on sharing protocols at . Additionally, PLOS ONE offers an option for publishing peer-reviewed Lab Protocol articles, which describe protocols hosted on protocols.io. Read more information on sharing protocols at . Additionally, PLOS ONE offers an option for publishing peer-reviewed Lab Protocol articles, which describe protocols hosted on protocols.io. Read more information on sharing protocols at . Additionally, PLOS ONE offers an option for publishing peer-reviewed Lab Protocol articles, which describe protocols hosted on protocols.io. Read more information on sharing protocols at https://plos.org/protocols?utm_medium=editorial-email&utm_source=authorletters&utm_campaign=protocols....

We look forward to receiving your revised manuscript.

Kind regards,

Agegnehu Bante

Academic Editor

PLOS ONE

Journal Requirements:

If the reviewer comments include a recommendation to cite specific previously published works, please review and evaluate these publications to determine whether they are relevant and should be cited. There is no requirement to cite these works unless the editor has indicated otherwise.

Please review your reference list to ensure that it is complete and correct. If you have cited papers that have been retracted, please include the rationale for doing so in the manuscript text, or remove these references and replace them with relevant current references. Any changes to the reference list should be mentioned in the rebuttal letter that accompanies your revised manuscript. If you need to cite a retracted article, indicate the article’s retracted status in the References list and also include a citation and full reference for the retraction notice.

[Note: HTML markup is below. Please do not edit.]

Reviewers' comments:

Reviewer's Responses to Questions

**Comments to the Author**

Reviewer #4: All comments have been addressed

Reviewer #6: (No Response)

**********

2. Is the manuscript technically sound, and do the data support the conclusions?

Reviewer #4: Yes

Reviewer #6: Partly

**********

3. Has the statistical analysis been performed appropriately and rigorously?

Reviewer #4: Yes

Reviewer #6: Yes

**********

4. Have the authors made all data underlying the findings in their manuscript fully available?

The PLOS Data policy requires authors to make all data underlying the findings described in their manuscript fully available without restriction, with rare exception (please refer to the Data Availability Statement in the manuscript PDF file). The data should be provided as part of the manuscript or its supporting information, or deposited to a public repository. For example, in addition to summary statistics, the data points behind means, medians and variance measures should be available. If there are restrictions on publicly sharing data—e.g. participant privacy or use of data from a third party—those must be specified.requires authors to make all data underlying the findings described in their manuscript fully available without restriction, with rare exception (please refer to the Data Availability Statement in the manuscript PDF file). The data should be provided as part of the manuscript or its supporting information, or deposited to a public repository. For example, in addition to summary statistics, the data points behind means, medians and variance measures should be available. If there are restrictions on publicly sharing data—e.g. participant privacy or use of data from a third party—those must be specified.

Reviewer #4: Yes

Reviewer #6: Yes

**********

5. Is the manuscript presented in an intelligible fashion and written in standard English?

Reviewer #4: Yes

Reviewer #6: Yes

**********

Reviewer #4: I appreciate the authors’ attention to responding effectively to the previous issues, resulting in a much-improved version of the manuscript. Despite these advancements, the paper is still hindered by grammatical errors, unclear sentence structures, and inconsistencies that detract from clarity and weaken its overall impact.

The Introduction effectively contextualizes the problem, defines key terms, and highlights the study’s relevance. While it is generally well-structured with a clear research gap and notable improvements in flow, it would benefit from minor polishing to address run-on sentences, redundancy, and enhance conciseness.

The Methods section is logically organized with adequate detail but requires further editing for grammar, formatting, and clarity. Addressing awkward phrasing, run-on sentences, and inconsistent tense usage will improve readability and academic rigor, ensuring the manuscript is publication-ready.

The Discussion is content-rich, appropriately identifies patterns, explains findings, and addresses limitations. However, it needs revision to improve grammar, sentence structure, and logical flow; eliminate redundancy; clarify contradictory points (notably regarding age versus parity); and strengthen the conclusion with actionable recommendations.

Overall, the manuscript demonstrates commendable attention to detail and effectively addresses the key issues previously raised. I commend the authors for their work and encourage a thorough final edit to resolve remaining grammatical issues, improve clarity, and ensure consistency for a polished, professional presentation.

Reviewer #6: The original reviewers note, correctly, that a 1 month study period is extremely short for a cross-sectional study design. The authors responded to this comment noting that the study period was actually 1 year and that detail was updated in the revised manuscript, however the abstract in the revised version clearly states only a 1 month study period from 30 June 2023 to 30 July 2023. Then in the Methods Section the authors note the longer time-period from 01 September 2021 - 30 August 2022, but it's unclear why they classify this as "secondary data" and how the data in the 1-month and 1-year study periods are related. Are all of these results included in the results section or only some? It's very unclear and should be updated.

The overall goal/conclusion of this study was to measure the effective ANC coverage for this population and the authors clearly define effective ANC coverage as "attended four or more antenatal care (ANC) visits, and they received all the WHO recommended interventions at least once during". Given this definition, it does not make sense that the authors conclude that the effective ANC coverage is 25.9%. How is this possible if Table 5 shows that only 8.2% of women received "Screening for gestational diabetes mellitus" which is one of the noted/required WHO interventions? Given this data and how the authors define effective coverage, wouldn't that mean only 8.2% met the standard for effective coverage? The breakdown in services and what is/isn't provided to pregnant individuals in this population is very interesting, but I think the data interpretation doesn't match what the authors themselves laid out as qualifying as effective coverage.

**********

what does this mean?). If published, this will include your full peer review and any attached files.). If published, this will include your full peer review and any attached files.). If published, this will include your full peer review and any attached files.). If published, this will include your full peer review and any attached files.

If you choose “no”, your identity will remain anonymous but your review may still be made public.

**Do you want your identity to be public for this peer review?** For information about this choice, including consent withdrawal, please see our For information about this choice, including consent withdrawal, please see our For information about this choice, including consent withdrawal, please see our For information about this choice, including consent withdrawal, please see our Privacy Policy..

Reviewer #4: No

Reviewer #6: **Yes:** Samantha A ByrnesSamantha A ByrnesSamantha A ByrnesSamantha A Byrnes

**********

[NOTE: If reviewer comments were submitted as an attachment file, they will be attached to this email and accessible via the submission site. Please log into your account, locate the manuscript record, and check for the action link "View Attachments". If this link does not appear, there are no attachment files.]

To ensure your figures meet our technical requirements, please review our figure guidelines: https://journals.plos.org/plosone/s/figures

You may also use PLOS’s free figure tool, NAAS, to help you prepare publication quality figures: https://journals.plos.org/plosone/s/figures#loc-tools-for-figure-preparation.

NAAS will assess whether your figures meet our technical requirements by comparing each figure against our figure specifications.

# Author comment: Author response to Decision Letter 3

**DOI:** 10.1371/journal.pone.0347341.r006

3 Nov 2025

Title: Effective Coverage and Associated Factors of Antenatal Care Service among Women Who Attended Antenatal Care at West Gojjam Zone Public Hospitals, Northwest Ethiopia

Reviewer's Responses to Questions

Comments to the Author

1. If the authors have adequately addressed your comments raised in a previous round of review and you feel that this manuscript is now acceptable for publication, you may indicate that here to bypass the “Comments to the Author” section, enter your conflict of interest statement in the “Confidential to Editor” section, and submit your "Accept" recommendation.

Reviewer #4: All comments have been addressed

Reviewer #6: (No Response)

Response: Thank you! We have tried to address the remaining comments in the revised manuscript.

2. Is the manuscript technically sound, and do the data support the conclusions?

The manuscript must describe a technically sound piece of scientific research with data that supports the conclusions. Experiments must have been conducted rigorously, with appropriate controls, replication, and sample sizes. The conclusions must be drawn appropriately based on the data presented.

Reviewer #4: Yes

Reviewer #6: Partly

Response: Thank you!

3. Has the statistical analysis been performed appropriately and rigorously?

Reviewer #4: Yes

Reviewer #6: Yes

Response: Thank you!

4. Have the authors made all data underlying the findings in their manuscript fully available?

The PLOS Data policy requires authors to make all data underlying the findings described in their manuscript fully available without restriction, with rare exception (please refer to the Data Availability Statement in the manuscript PDF file). The data should be provided as part of the manuscript or its supporting information, or deposited to a public repository. For example, in addition to summary statistics, the data points behind means, medians and variance measures should be available. If there are restrictions on publicly sharing data—e.g. participant privacy or use of data from a third party—those must be specified.

Reviewer #4: Yes

Reviewer #6: Yes

Response: Thank you!

5. Is the manuscript presented in an intelligible fashion and written in standard English?

PLOS ONE does not copyedit accepted manuscripts, so the language in submitted articles must be clear, correct, and unambiguous. Any typographical or grammatical errors should be corrected at revision, so please note any specific errors here.

Reviewer #4: Yes

Reviewer #6: Yes

Response: Thank you!

6. Review Comments to the Author

Please use the space provided to explain your answers to the questions above. You may also include additional comments for the author, including concerns about dual publication, research ethics, or publication ethics. (Please upload your review as an attachment if it exceeds 20,000 characters)

Reviewer #4: I appreciate the authors’ attention to responding effectively to the previous issues, resulting in a much-improved version of the manuscript. Despite these advancements, the paper is still hindered by grammatical errors, unclear sentence structures, and inconsistencies that detract from clarity and weaken its overall impact.

Response: we really appreciated the raised comments and as much as possible we have tried a lot to correct the grammatical errors in the revised version of the manuscript.

The Introduction effectively contextualizes the problem, defines key terms, and highlights the study’s relevance. While it is generally well-structured with a clear research gap and notable improvements in flow, it would benefit from minor polishing to address run-on sentences, redundancy, and enhance conciseness.

Response: Thank you! It is very important comment and we have corrected the introduction section accordingly.

The Methods section is logically organized with adequate detail but requires further editing for grammar, formatting, and clarity. Addressing awkward phrasing, run-on sentences, and inconsistent tense usage will improve readability and academic rigor, ensuring the manuscript is publication-ready.

Response: Thank you! It is very important comment and we have corrected the whole method sections accordingly.

The Discussion is content-rich, appropriately identifies patterns, explains findings, and addresses limitations. However, it needs revision to improve grammar, sentence structure, and logical flow; eliminate redundancy; clarify contradictory points (notably regarding age versus parity); and strengthen the conclusion with actionable recommendations.

Response: we really wonder this issue. It is corrected accordingly. See the revised manuscript.

Overall, the manuscript demonstrates commendable attention to detail and effectively addresses the key issues previously raised. I commend the authors for their work and encourage a thorough final edit to resolve remaining grammatical issues, improve clarity, and ensure consistency for a polished, professional presentation.

Response: Thank you! It is very important comments and we corrected the revised manuscript accordingly.

Reviewer #6: The original reviewers note, correctly, that a 1 month study period is extremely short for a cross-sectional study design. The authors responded to this comment noting that the study period was actually 1 year and that detail was updated in the revised manuscript, however the abstract in the revised version clearly states only a 1 month study period from 30 June 2023 to 30 July 2023. Then in the Methods Section the authors note the longer time-period from 01 September 2021 - 30 August 2022, but it's unclear why they classify this as "secondary data" and how the data in the 1-month and 1-year study periods are related. Are all of these results included in the results section or only some? It's very unclear and should be updated.

Response: we really appreciated the concern. This study is a facility based cross- sectional study. The data were collected from pregnant women ANC follow up chart (those pregnant women who had ANC follow up at study area public hospitals from 01 September 2021 - 30 August 2022). Even if the study period was a year i.e. from 01 September 2021 - 30 August 2022, we extracted the data from pregnant mothers ANC follow up chart from 30 June 2023 to 30 July 2023 (a month). Therefore, the study period and actual data extraction/collection period were different, i.e. why we put the two time period here. This study uses secondary data because the data were extracted from clients chart, not directly collected from study participants.

The overall goal/conclusion of this study was to measure the effective ANC coverage for this population and the authors clearly define effective ANC coverage as "attended four or more antenatal care (ANC) visits, and they received all the WHO recommended interventions at least once during". Given this definition, it does not make sense that the authors conclude that the effective ANC coverage is 25.9%. How is this possible if Table 5 shows that only 8.2% of women received "Screening for gestational diabetes mellitus" which is one of the noted/required WHO interventions? Given this data and how the authors define effective coverage, wouldn't that mean only 8.2% met the standard for effective coverage? The breakdown in services and what is/isn't provided to pregnant individuals in this population is very interesting, but I think the data interpretation doesn't match what the authors themselves laid out as qualifying as effective coverage.

Response: Really, pleased comment. We were confused while we see this comment and we have checked our data set from the beginning to the end and we found that 8.2 % were “Screening for gestational diabetes mellitus” at each visit as we put in the table 5 of the revised manuscript ( line 255). But the pregnant women who had received “Screening for gestational diabetes mellitus at least once was 25.88%, see the revised manuscript” and this inconvenience was occurred during manuscript preparation. As you know, to conduct statistical analysis determining the outcome variable status is first above all. We were done that considering the above figure (25.88%) for “screening for gestational diabetes mellitus’’ at least once. Lastly, we appreciated a lot in this regard since it is very crucial for the thoroughness of our manuscript.

Generally, we feel that your valuable comments and insights make our manuscript well improved and we are too happy due to getting such very important inputs.

Attachment Submitted filename: Point by point response letter 3.docx

# Peer review: Decision Letter 3

**DOI:** 10.1371/journal.pone.0347341.r007

**Authors:** Agegnehu Bante

22 Feb 2026

Dear Dr. Endalew,

Thank you for submitting your manuscript to PLOS ONE. After careful consideration, we feel that it has merit but does not fully meet PLOS ONE’s publication criteria as it currently stands. Therefore, we invite you to submit a revised version of the manuscript that addresses the points raised during the review process.

Please submit your revised manuscript by April 5, 2026, 11:59. If you will need more time than this to complete your revisions, please reply to this message or contact the journal office at plosone@plos.org. When you're ready to submit your revision, log on to https://www.editorialmanager.com/pone/ and select the 'Submissions Needing Revision' folder to locate your manuscript file.. When you're ready to submit your revision, log on to https://www.editorialmanager.com/pone/ and select the 'Submissions Needing Revision' folder to locate your manuscript file.. When you're ready to submit your revision, log on to https://www.editorialmanager.com/pone/ and select the 'Submissions Needing Revision' folder to locate your manuscript file.. When you're ready to submit your revision, log on to https://www.editorialmanager.com/pone/ and select the 'Submissions Needing Revision' folder to locate your manuscript file.

- A letter that responds to each point raised by the academic editor and reviewer(s). You should upload this letter as a separate file labeled 'Response to Reviewers'.
- A marked-up copy of your manuscript that highlights changes made to the original version. You should upload this as a separate file labeled 'Revised Manuscript with Track Changes'.
- An unmarked version of your revised paper without tracked changes. You should upload this as a separate file labeled 'Manuscript'.

If applicable, we recommend that you deposit your laboratory protocols in protocols.io to enhance the reproducibility of your results. Protocols.io assigns your protocol its own identifier (DOI) so that it can be cited independently in the future. For instructions see: https://journals.plos.org/plosone/s/submission-guidelines#loc-laboratory-protocols. Additionally, PLOS ONE offers an option for publishing peer-reviewed Lab Protocol articles, which describe protocols hosted on protocols.io. Read more information on sharing protocols at . Additionally, PLOS ONE offers an option for publishing peer-reviewed Lab Protocol articles, which describe protocols hosted on protocols.io. Read more information on sharing protocols at . Additionally, PLOS ONE offers an option for publishing peer-reviewed Lab Protocol articles, which describe protocols hosted on protocols.io. Read more information on sharing protocols at . Additionally, PLOS ONE offers an option for publishing peer-reviewed Lab Protocol articles, which describe protocols hosted on protocols.io. Read more information on sharing protocols at https://plos.org/protocols?utm_medium=editorial-email&utm_source=authorletters&utm_campaign=protocols....

We look forward to receiving your revised manuscript.

Kind regards,

Agegnehu Bante

Academic Editor

PLOS One

Journal Requirements:

If the reviewer comments include a recommendation to cite specific previously published works, please review and evaluate these publications to determine whether they are relevant and should be cited. There is no requirement to cite these works unless the editor has indicated otherwise.

Please review your reference list to ensure that it is complete and correct. If you have cited papers that have been retracted, please include the rationale for doing so in the manuscript text, or remove these references and replace them with relevant current references. Any changes to the reference list should be mentioned in the rebuttal letter that accompanies your revised manuscript. If you need to cite a retracted article, indicate the article’s retracted status in the References list and also include a citation and full reference for the retraction notice.

[Note: HTML markup is below. Please do not edit.]

Reviewers' comments:

Reviewer's Responses to Questions

**Comments to the Author**

Reviewer #7: All comments have been addressed

Reviewer #8: All comments have been addressed

**********

2. Is the manuscript technically sound, and do the data support the conclusions?

Reviewer #7: Yes

Reviewer #8: Yes

**********

3. Has the statistical analysis been performed appropriately and rigorously?

Reviewer #7: Yes

Reviewer #8: Yes

**********

4. Have the authors made all data underlying the findings in their manuscript fully available?

The PLOS Data policy requires authors to make all data underlying the findings described in their manuscript fully available without restriction, with rare exception (please refer to the Data Availability Statement in the manuscript PDF file). The data should be provided as part of the manuscript or its supporting information, or deposited to a public repository. For example, in addition to summary statistics, the data points behind means, medians and variance measures should be available. If there are restrictions on publicly sharing data—e.g. participant privacy or use of data from a third party—those must be specified.requires authors to make all data underlying the findings described in their manuscript fully available without restriction, with rare exception (please refer to the Data Availability Statement in the manuscript PDF file). The data should be provided as part of the manuscript or its supporting information, or deposited to a public repository. For example, in addition to summary statistics, the data points behind means, medians and variance measures should be available. If there are restrictions on publicly sharing data—e.g. participant privacy or use of data from a third party—those must be specified.

Reviewer #7: Yes

Reviewer #8: Yes

**********

5. Is the manuscript presented in an intelligible fashion and written in standard English?

Reviewer #7: Yes

Reviewer #8: Yes

**********

Reviewer #7: Reviewer Comments for Manuscript PONE-D-24-45499R3

This manuscript contains grammatical errors, awkward phrasing, and inconsistent tense usage that reduce clarity and readability. Several sentences are overly long or redundant, which weakens the logical flow of the argument. I recommend a thorough language edit to:

• Correct grammar and typographical errors.

• Ensure consistent use of past tense in the Methods and Results sections, and present tense in the Introduction and Discussion…(e.g., “effective coverage,” “crude coverage”)

• Shorten or restructure overly long sentences to improve conciseness and readability. Minor typographical errors and redundancies should be carefully polished before publication.

Suggestion: The manuscript would benefit significantly from thorough proofreading by a native English speaker or professional editing service to improve clarity and adherence to academic writing standards.

2. Abstract

• The abstract is informative but needs refinement for clarity.

• The study period is confusing: the abstract mentions June–July 2023 (1 month), while the methods section refers to September 2021–August 2022 (1 year). This inconsistency must be corrected. You have to remove … “using secondary data of mothers who received antenatal care from September 01, 2021, to August 30, 2022”. You have also change design into “retrospective cross-sectional studies”. Change…”bi variable logistic regression” → “bivariable logistic regression”;

• The results section of the abstract should align with the operational definition of effective coverage. For example, if screening for gestational diabetes is required, how does the reported 25.9% coverage reconcile with only 8.2% receiving that service?

3. Introduction

• The introduction is well contextualized, but some sentences are repetitive and could be streamlined.

• The rationale for focusing on effective coverage rather than crude coverage is well stated, but the flow could be improved by reducing redundancy.

• Consider clarifying the conceptual framework of effective coverage (need, use, quality) more succinctly.

• Suggestion: Consider adding a sentence on why West Gojjam Zone specifically was chosen—e.g., known gaps in ANC quality, high maternal mortality, or representative setting.

• Question: The authors state that “none have examined EC of ANC in our region especially in my study setting.” Were there any sub-national or facility-based ANC quality assessments that could be referenced for context?

Methods

Study design and period

The description of the study design is somewhat confusing. The manuscript currently states: “A facility-based analytical cross-sectional study design was conducted in West Gojjam public hospitals from June 30 to July 30, 2023, utilizing secondary data from mothers who received antenatal care between September 1, 2021, and August 30, 2022.”

• Since the data were extracted retrospectively from ANC charts, the design would be more accurately described as a retrospective cross-sectional study rather than “analytical cross-sectional.”

• The phrase “utilizing secondary data from mothers who received antenatal care between September 1, 2021, and August 30, 2022” is redundant here, because the study population and source of data are already explained elsewhere.

• I recommend that the authors simplify this section to report only the study design and data collection period, while clearly noting that secondary data were used. For example: “A facility-based retrospective cross-sectional study was conducted in West Gojjam public hospitals. Data were collected between June 30 and July 30, 2023, from ANC charts covering the period September 1, 2021, to August 30, 2022.”

Sampling Size Calculation

I appreciate that the authors calculated the sample size for both objectives (coverage and associated factors). However, including the full formula in the manuscript is not necessary, since you have already stated that the single population proportion formula was used.

I recommend simplifying this section by:

• Clearly stating that the sample size was calculated for both objectives.

• Indicating that the largest sample size obtained was selected as the final sample size for the study.

• Removing the redundant formula presentation, while keeping the assumptions (confidence level, margin of error, prevalence, design effect, non-response rate) clearly described.

This will make the Methods section more concise and reader friendly, while still transparent about how the sample size was determined.

Study Design and Sampling:

• The multi-stage random sampling is appropriate, but the description of selecting 3 out of 8 hospitals (38%) by “lottery” is vague. Were all hospitals eligible? Was stratification considered?

• Suggestion: Clarify whether the selection was truly random or purposive, and justify the sample size allocation.

Why were only 3 hospitals selected? Was power considered at the hospital level?

Why was a design effect of 1.5 applied? Was clustering expected, and if so, at what level? Please justify this choice.

Measurement of Effective Coverage:

The definition aligns with WHO recommendations, but the operationalization—“received all the WHO recommended interventions at least once”—may overlook frequency and timing of interventions, which is acknowledged as a limitation.

Suggestion: Briefly note how missing data on intervention timing was handled.

How was “adequately trained human power” assessed? Was it based on presence per shift, or per patient load?

Clarify whether data were extracted only from charts or supplemented with interviews.

Line 181-182. Avoid----�remove…”The questionnaire was prepared in English and

182 pretested among 5% (34) of study population before the actual data collection”…this repetition/The author already included in data quality control.

Data Analysis:

The use of p<0.25 for inclusion in multivariable analysis is acceptable but should be justified given the sample size.

Suggestion: Consider also using variance inflation factors (VIF) to check multicollinearity, especially among obstetric/gynecological variables.

Question: Why were hospital-level factors (e.g., equipment, staffing) not included in the regression model?

Results:

Tables are comprehensive but could be better formatted. For example, Table 5 is split across pages Comment on Definition and Results of Effective Coverage

The manuscript defines effective coverage of ANC interventions as: pregnant women who attended four or more ANC visits and received all WHO recommended interventions at least once during their ANC follow up period.

However, the results presented raise important concerns:

• For several interventions, the proportion of women receiving them “at least once” is far below 25.9%. For example, only 8.2% received antenatal ultrasound and 8.2% were screened for gestational diabetes mellitus.

• If all WHO recommended interventions are required for effective coverage, then the reported 25.9% effective coverage seems inconsistent with these low uptake rates.

• The table also mixes “at each visit” and “at least once” indicators, which makes interpretation difficult. For example, gestational diabetes screening is reported as 25.9% “at each visit” but only 8.2% “at least once.” This needs clarification.

• The operational definition should be applied consistently: if “all WHO interventions at least once” is the criterion, then the effective coverage proportion should reflect the lowest uptake intervention (e.g., 8.2%).

Recommendations:

1. Clarify the operational definition of effective coverage — is it “all WHO interventions at least once” or “some interventions at least once”?

2. Ensure consistency between the definition and the reported results.

3. Revise the Results section to avoid contradictory figures (e.g., 25.9% vs. 8.2%).

4. Consider presenting both crude coverage (ANC 4+) and effective coverage (ANC 4+ plus all WHO interventions) separately, with clear explanation.

Discussion:

The discussion appropriately contextualizes findings within existing literature and offers plausible explanations for discrepancies.

To strengthen the impact of your manuscript, please revise the Discussion to move beyond restating the Results. Instead, focus on interpreting the clinical and public health significance of your key findings. For each major result, please address:

1. What does this mean for practice? (e.g., How should the very low rate of Gestational Diabetes Mellitus screening change clinical protocols or health worker training?)

2. What are the policy implications? (e.g., Does the finding that women with prior complications receive better coverage suggest a need to reallocate resources or change messaging to reach low-risk women?)

3. How can these findings inform action? Conclude with targeted, practical recommendations for health system managers and policymakers in your setting."

Question: The finding that prior complications increase effective coverage suggests that risk-based care is occurring. Does this imply that low-risk women are being neglected? This has important programmatic implications.

Reviewer #8: (No Response)

**********

what does this mean?). If published, this will include your full peer review and any attached files.). If published, this will include your full peer review and any attached files.). If published, this will include your full peer review and any attached files.). If published, this will include your full peer review and any attached files.

If you choose “no”, your identity will remain anonymous but your review may still be made public.

**Do you want your identity to be public for this peer review?** For information about this choice, including consent withdrawal, please see our For information about this choice, including consent withdrawal, please see our For information about this choice, including consent withdrawal, please see our For information about this choice, including consent withdrawal, please see our Privacy Policy..

Reviewer #7: No

Reviewer #8: No

**********

[NOTE: If reviewer comments were submitted as an attachment file, they will be attached to this email and accessible via the submission site. Please log into your account, locate the manuscript record, and check for the action link "View Attachments". If this link does not appear, there are no attachment files.]

To ensure your figures meet our technical requirements, please review our figure guidelines: https://journals.plos.org/plosone/s/figures

You may also use PLOS’s free figure tool, NAAS, to help you prepare publication quality figures: https://journals.plos.org/plosone/s/figures#loc-tools-for-figure-preparation.

NAAS will assess whether your figures meet our technical requirements by comparing each figure against our figure specifications.

# Author comment: Author response to Decision Letter 4

**DOI:** 10.1371/journal.pone.0347341.r008

10 Mar 2026

Point by point response

Title: Effective Coverage and Associated Factors of Antenatal Care Service among Women Who Attended Antenatal Care at West Gojjam Zone Public Hospitals, Northwest Ethiopia

Comments to the Author

1. If the authors have adequately addressed your comments raised in a previous round of review and you feel that this manuscript is now acceptable for publication, you may indicate that here to bypass the “Comments to the Author” section, enter your conflict of interest statement in the “Confidential to Editor” section, and submit your "Accept" recommendation.

Reviewer #7: All comments have been addressed

Reviewer #8: All comments have been addressed

2. Is the manuscript technically sound, and do the data support the conclusions?

The manuscript must describe a technically sound piece of scientific research with data that supports the conclusions. Experiments must have been conducted rigorously, with appropriate controls, replication, and sample sizes. The conclusions must be drawn appropriately based on the data presented.

Reviewer #7: Yes

Reviewer #8: Yes

3. Has the statistical analysis been performed appropriately and rigorously?

Reviewer #7: Yes

Reviewer #8: Yes

4. Have the authors made all data underlying the findings in their manuscript fully available?

The PLOS Data policy requires authors to make all data underlying the findings described in their manuscript fully available without restriction, with rare exception (please refer to the Data Availability Statement in the manuscript PDF file). The data should be provided as part of the manuscript or its supporting information, or deposited to a public repository. For example, in addition to summary statistics, the data points behind means, medians and variance measures should be available. If there are restrictions on publicly sharing data—e.g. participant privacy or use of data from a third party those must be specified.

Reviewer #7: Yes

Reviewer #8: Yes

5. Is the manuscript presented in an intelligible fashion and written in standard English?

PLOS ONE does not copyedit accepted manuscripts, so the language in submitted articles must be clear, correct, and unambiguous. Any typographical or grammatical errors should be corrected at revision, so please note any specific errors here.

Reviewer #7: Yes

Reviewer #8: Yes

6. Review Comments to the Author

Please use the space provided to explain your answers to the questions above. You may also include additional comments for the author, including concerns about dual publication, research ethics, or publication ethics. (Please upload your review as an attachment if it exceeds 20,000 characters)

Reviewer #7: Reviewer Comments for Manuscript PONE-D-24-45499R3

1. This manuscript contains grammatical errors, awkward phrasing, and inconsistent tense usage that reduce clarity and readability. Several sentences are overly long or redundant, which weakens the logical flow of the argument. I recommend a thorough language edit to: Correct grammar and typographical errors

Response: We really appreciate your concern and we have seen thoroughly the whole and document and corrected accordingly. See the revised manuscript.

• Ensure consistent use of past tense in the Methods and Results sections, and present tense in the Introduction and Discussion…(e.g., “effective coverage,” “crude coverage”)

Response: It is interesting and we corrected it accordingly.

• Shorten or restructure overly long sentences to improve conciseness and readability. Minor typographical errors and redundancies should be carefully polished before publication.

Response: corrected accordingly.

Suggestion: The manuscript would benefit significantly from thorough proofreading by a native English speaker or professional editing service to improve clarity and adherence to academic writing standards.

Response: we will be happy if it is done.

2. Abstract

• The abstract is informative but needs refinement for clarity.

Response: Thank you. We have tried to correct as per your suggestion.

• The study period is confusing: the abstract mentions June–July 2023 (1 month), while the methods section refers to September 2021–August 2022 (1 year). This inconsistency must be corrected. You have to remove … “using secondary data of mothers who received antenatal care from September 01, 2021, to August 30, 2022”.

Response: corrected. See line 103 to 105 of the revised manuscript.

You have also change design into “retrospective cross-sectional studies”

Response: really, corrected accordingly.

Change…”bi variable logistic regression” → “bivariable logistic regression”;

Response: corrected.

• The results section of the abstract should align with the operational definition of effective coverage. For example, if screening for gestational diabetes is required, how does the reported 25.9% coverage reconcile with only 8.2% receiving that service?

Response: It is very interesting concern and corrected accordingly. See the revised manuscript table 4.

3. Introduction

• The introduction is well contextualized, but some sentences are repetitive and could be streamlined.

Response: corrected accordingly

• The rationale for focusing on effective coverage rather than crude coverage is well stated, but the flow could be improved by reducing redundancy.

Response: corrected accordingly

• Consider clarifying the conceptual framework of effective coverage (need, use, quality) more succinctly.

Response: corrected accordingly

• Suggestion: Consider adding a sentence on why West Gojjam Zone specifically was chosen—e.g., known gaps in ANC quality, high maternal mortality, or representative setting.

Response: we tried to add evidence in this regard. See the revised manuscript.

• Question: The authors state that “none have examined EC of ANC in our region especially in my study setting.” Were there any sub-national or facility-based ANC quality assessments that could be referenced for context?

Response: corrected accordingly.

Methods

Study design and period

The description of the study design is somewhat confusing. The manuscript currently states: “A facility-based analytical cross-sectional study design was conducted in West Gojjam public hospitals from June 30 to July 30, 2023, utilizing secondary data from mothers who received antenatal care between September 1, 2021, and August 30, 2022.”

Response: corrected accordingly, see line 103 to 105 of the revised manuscript.

• Since the data were extracted retrospectively from ANC charts, the design would be more accurately described as a retrospective cross-sectional study rather than “analytical cross-sectional.”

Response: Really, it should be.

• The phrase “utilizing secondary data from mothers who received antenatal care between September 1, 2021, and August 30, 2022” is redundant here, because the study population and source of data are already explained elsewhere.

Response: The redundancy is avoided. See the revised manuscript.

• I recommend that the authors simplify this section to report only the study design and data collection period, while clearly noting that secondary data were used. For example: “A facility-based retrospective cross-sectional study was conducted in West Gojjam public hospitals. Data were collected between June 30 and July 30, 2023, from ANC charts covering the period September 1, 2021, to August 30, 2022.”

Response: corrected accordingly

Sampling Size Calculation

I appreciate that the authors calculated the sample size for both objectives (coverage and associated factors). However, including the full formula in the manuscript is not necessary, since you have already stated that the single population proportion formula was used.

I recommend simplifying this section by: Clearly stating that the sample size was calculated for both objectives. Indicating that the largest sample size obtained was selected as the final sample size for the study. Removing the redundant formula presentation, while keeping the assumptions (confidence level, margin of error, prevalence, design effect, non-response rate) clearly described. This will make the Methods section more concise and reader friendly, while still transparent about how the sample size was determined.

Response: corrected accordingly. See the revised manuscript line 130 to 137.

Study Design and Sampling:

• The multi-stage random sampling is appropriate, but the description of selecting 3 out of 8 hospitals (38%) by “lottery” is vague. Were all hospitals eligible? Was stratification considered?

• Suggestion: Clarify whether the selection was truly random or purposive, and justify the sample size allocation.

Response: Thank you for the valuable comments regarding the sampling procedure. We have clarified the sampling approach as follows: All eight public hospitals in West Gojjam Zone were eligible for inclusion. There were no exclusion criteria. Since the hospitals are relatively homogeneous in terms of service structure, level of care (general and primary hospitals), and provision of antenatal care services under the same regional guidelines, stratification was not considered necessary. The selection of three hospitals was conducted using a simple random sampling (lottery) method. The names of all eight hospitals were written on separate pieces of paper, mixed thoroughly, and three were drawn randomly. Therefore, the selection was entirely random and not purposive.

Why were only 3 hospitals selected? Was power considered at the hospital level?

Response: The number of hospitals selected was determined primarily based on feasibility, logistics, and available resources and the recommended selection of study site is 20 to 30% and we took 30 % (the maximum) and 8*30%=2.4 which is approximate to 3: still ensuring representativeness of the zone. The primary unit of analysis in this study was the individual mother (not the hospital). The sample size calculation was based on estimating the prevalence of effective ANC coverage at the individual level. Therefore, statistical power was calculated at the participant level rather than the hospital level. The study was not powered to detect differences between hospitals. Why was a design effect of 1.5 applied? Was clustering expected, and if so, at what level? Please justify this choice.

Response: A design effect of 1.5 was applied to account for potential clustering at the hospital level, as participants were nested within selected hospitals. Although individual-level simple random sampling was used within each hospital, responses may be correlated due to shared service environment, provider practices, and institutional characteristics. Since prior intra-cluster correlation (ICC) estimates for effective ANC coverage in similar settings were not available, a conservative design effect of 1.5 was used, which is commonly applied in health service research conducted in similar contexts. This adjustment helped to maintain adequate precision despite possible clustering. https://www.google.com/search?q=DESIGNING+AND+SELECTING+THE+SAMPLE&oq=DESIGNING+AND+SELECTING+THE+SAMPLE&gs_lcrp=EgZjaHJvbWUyBggAEEUYOTIICAEQABgWGB4yBwgCEAAY7wUyCggDEAAYgAQYogQyBwgEEAAY7wUyCggFEAAYgAQYogQyCggGEAAYgAQYogTSAQkxODc0ajBqMTWoAgywAgHxBWtFjwtMWGeG&sourceid=chrome&ie=UTF-8

Measurement of Effective Coverage:

The definition aligns with WHO recommendations, but the operationalization—“received all the WHO recommended interventions at least once”may overlook frequency and timing of interventions, which is acknowledged as a limitation.

Response: Thank you, we have considered it as a limitation in the revised manuscript.

Suggestion: Briefly note how missing data on intervention timing was handled.

How was “adequately trained human power” assessed? Was it based on presence per shift, or per patient load?

Response: it was done per patient load.

Clarify whether data were extracted only from charts or supplemented with interviews.

Response: only chart review.

Line 181-182. Avoid----�remove…”The questionnaire was prepared in English and

182 pretested among 5% (34) of study population before the actual data collection”…this repetition/The author already included in data quality control.

Response: done.

Data Analysis:

The use of p<0.25 for inclusion in multivariable analysis is acceptable but should be justified given the sample size.

Response: The use of p < 0.25 as a screening criterion for inclusion in the multivariable logistic regression model was guided by methodological recommendations in regression modeling. This relatively liberal threshold is commonly applied at the bivariable stage to avoid excluding potentially important variables that may not show strong crude associations but could become significant after adjustment for confounding. Given our total sample size of n = 680, the study had adequate power to accommodate this approach. The number of outcome events was sufficient to maintain an acceptable events-per-variable (EPV) ratio in the final multivariable model. To prevent over fitting, we ensured that: only theoretically plausible variables were considered, the final model satisfied EPV assumptions (≥10 events per predictor variable)and multicollinearity was assessed before model fitting. Therefore, using p < 0.25 at the bivariable level allowed us to reduce the risk of residual confounding while maintaining model stability and statistical validity.

Suggestion: Consider also using variance inflation factors (VIF) to check multicollinearity, especially among obstetric/gynecological variables.

Response: multicollinearity was checked before fitting the final model of regression using variance inflation factor and tolerance test and there was no correlation b/n independent factors.

Question: Why were hospital-level factors (e.g., equipment, staffing) not included in the regression model?

Response: Hospital-level variables were not included because the study focused on individual-level determinants, only three hospitals were included (insufficient for stable cluster-level modeling), and facilities were structurally similar under the same regional standards. Future research using larger numbers of hospitals and multilevel analysis is recommended.

Results:

Tables are comprehensive but could be better formatted. For example, Table 5 is split across pages Comment on Definition and Results of Effective Coverage

The manuscript defines effective coverage of ANC interventions as: pregnant women who attended four or more ANC visits and received all WHO recommended interventions at least once during their ANC follow up period. However, the results presented raise important concerns: For several interventions, the proportion of women receiving them “at least once” is far below 25.9%. For example, only 8.2% received antenatal ultrasound and 8.2% were screened for gestational diabetes mellitus. If all WHO recommended interventions are required for effective coverage, then the reported 25.9% effective coverage seems inconsistent with these low uptake rates.

Response: the operational definition and the result are consistent as per our research work. See table 4 of the revised manuscript. We tried to make it clear.

• The table also mixes “at each visit” and “at least once” indicators, which makes interpretation difficult. For example, gestational diabetes screening is reported as 25.9% “at each visit” but only 8.2% “at least once.” This needs clarification.

Response: see table 4 of the revised manuscript. We tried to make it clear. 25. 9% were screened for GDM at least once and 8.2% were screened for GDM at each visit and the Effective coverage was measured ANC4+ and WHO recommended services at least once. Hence, there is no inconsistence result here.

• The operational definition should be applied consistently: if “all WHO interventions at least once” is the criterion, then the effective coverage proportion should reflect the lowest uptake intervention (e.g., 8.2%).

Response: this is the least proportion for all recommended services give at each visit and not considered as a measure of Effective coverage.

Recommendations:

1. Clarify the operational definition of effective coverage — is it “all WHO interventions at least once” or “some interventions at least once”?

Response: All intervention at least on

Attachment Submitted filename: point by point response.docx

# Peer review: Decision Letter 4

**DOI:** 10.1371/journal.pone.0347341.r009

**Authors:** Agegnehu Bante

31 Mar 2026

<p>Effective Coverage and Associated Factors of Antenatal Care Service among Women Who Attended Antenatal Care at West Gojjam Zone Public Hospitals, Northwest Ethiopia

PONE-D-24-45499R4

Dear Mr. Enalew,

We’re pleased to inform you that your manuscript has been judged scientifically suitable for publication and will be formally accepted for publication once it meets all outstanding technical requirements.

Within one week, you’ll receive an e-mail detailing the required amendments. When these have been addressed, you’ll receive a formal acceptance letter and your manuscript will be scheduled for publication.

An invoice will be generated when your article is formally accepted. Please note, if your institution has a publishing partnership with PLOS and your article meets the relevant criteria, all or part of your publication costs will be covered. Please make sure your user information is up-to-date by logging into Editorial Manager at Editorial Manager® and clicking the ‘Update My Information' link at the top of the page. For questions related to billing, please contact  and clicking the ‘Update My Information' link at the top of the page. For questions related to billing, please contact  and clicking the ‘Update My Information' link at the top of the page. For questions related to billing, please contact  and clicking the ‘Update My Information' link at the top of the page. For questions related to billing, please contact billing support....

If your institution or institutions have a press office, please notify them about your upcoming paper to help maximize its impact. If they’ll be preparing press materials, please inform our press team as soon as possible -- no later than 48 hours after receiving the formal acceptance. Your manuscript will remain under strict press embargo until 2 pm Eastern Time on the date of publication. For more information, please contact onepress@plos.org.

Kind regards,

Agegnehu Bante

Academic Editor

PLOS One

Additional Editor Comments (optional):

Reviewers' comments:

Reviewer's Responses to Questions

**Comments to the Author**

Reviewer #7: All comments have been addressed

Reviewer #8: All comments have been addressed

**********

2. Is the manuscript technically sound, and do the data support the conclusions?

Reviewer #7: Yes

Reviewer #8: Yes

**********

3. Has the statistical analysis been performed appropriately and rigorously?

Reviewer #7: Yes

Reviewer #8: Yes

**********

4. Have the authors made all data underlying the findings in their manuscript fully available?

The PLOS Data policy requires authors to make all data underlying the findings described in their manuscript fully available without restriction, with rare exception (please refer to the Data Availability Statement in the manuscript PDF file). The data should be provided as part of the manuscript or its supporting information, or deposited to a public repository. For example, in addition to summary statistics, the data points behind means, medians and variance measures should be available. If there are restrictions on publicly sharing data—e.g. participant privacy or use of data from a third party—those must be specified.requires authors to make all data underlying the findings described in their manuscript fully available without restriction, with rare exception (please refer to the Data Availability Statement in the manuscript PDF file). The data should be provided as part of the manuscript or its supporting information, or deposited to a public repository. For example, in addition to summary statistics, the data points behind means, medians and variance measures should be available. If there are restrictions on publicly sharing data—e.g. participant privacy or use of data from a third party—those must be specified.

Reviewer #7: Yes

Reviewer #8: Yes

**********

5. Is the manuscript presented in an intelligible fashion and written in standard English?

Reviewer #7: Yes

Reviewer #8: Yes

**********

Reviewer #7: (No Response)

Reviewer #8: (No Response)

**********

what does this mean?). If published, this will include your full peer review and any attached files.). If published, this will include your full peer review and any attached files.). If published, this will include your full peer review and any attached files.). If published, this will include your full peer review and any attached files.

If you choose “no”, your identity will remain anonymous but your review may still be made public.

**Do you want your identity to be public for this peer review?** For information about this choice, including consent withdrawal, please see our For information about this choice, including consent withdrawal, please see our For information about this choice, including consent withdrawal, please see our For information about this choice, including consent withdrawal, please see our Privacy Policy..

Reviewer #7: No

Reviewer #8: No

**********

# Editor report: Acceptance letter

**DOI:** 10.1371/journal.pone.0347341.r010

**Authors:** Agegnehu Bante

PONE-D-24-45499R4

PLOS One

Dear Dr. Endalew,

I'm pleased to inform you that your manuscript has been deemed suitable for publication in PLOS One. Congratulations! Your manuscript is now being handed over to our production team.

At this stage, our production department will prepare your paper for publication. This includes ensuring the following:

* All references, tables, and figures are properly cited

* All relevant supporting information is included in the manuscript submission,

* There are no issues that prevent the paper from being properly typeset

You will receive further instructions from the production team, including instructions on how to review your proof when it is ready. Please keep in mind that we are working through a large volume of accepted articles, so please give us a few days to review your paper and let you know the next and final steps.

Lastly, if your institution or institutions have a press office, please let them know about your upcoming paper now to help maximize its impact. If they'll be preparing press materials, please inform our press team within the next 48 hours. Your manuscript will remain under strict press embargo until 2 pm Eastern Time on the date of publication. For more information, please contact onepress@plos.org.

You will receive an invoice from PLOS for your publication fee after your manuscript has reached the completed accept phase. If you receive an email requesting payment before acceptance or for any other service, this may be a phishing scheme. Learn how to identify phishing emails and protect your accounts at https://explore.plos.org/phishing.

If we can help with anything else, please email us at customercare@plos.org.

Thank you for submitting your work to PLOS ONE and supporting open access.

Kind regards,

PLOS ONE Editorial Office Staff

on behalf of

Mr. Agegnehu Bante

Academic Editor

PLOS One
